# Ultrafast and hypersensitive phase imaging of propagating internodal current flows in myelinated axons and electromagnetic pulses in dielectrics

**DOI:** 10.1038/s41467-022-33002-8

**Published:** 2022-09-06

**Authors:** Yide Zhang, Binglin Shen, Tong Wu, Jerry Zhao, Joseph C. Jing, Peng Wang, Kanomi Sasaki-Capela, William G. Dunphy, David Garrett, Konstantin Maslov, Weiwei Wang, Lihong V. Wang

**Affiliations:** 1 Caltech Optical Imaging Laboratory, Andrew and Peggy Cherng Department of Medical Engineering, Department of Electrical Engineering, California Institute of Technology, Pasadena, CA 91125 USA; 2 Division of Biology and Biological Engineering, California Institute of Technology, Pasadena, CA 91125 USA; 3 Department of Biophysics, University of Texas Southwestern Medical Center, Dallas, TX 75390 USA; 4 Present Address: Key Laboratory of Optoelectronic Devices and Systems of Guangdong Province and Ministry of Education, College of Physics and Optoelectronic Engineering, Shenzhen University, Shenzhen, 518060 China; 5 Present Address: Key Laboratory of Space Photoelectric Detection and Perception, Nanjing University of Aeronautics and Astronautics, Nanjing, 210016 China

**Keywords:** Myelin biology and repair, Imaging and sensing, Phase-contrast microscopy

## Abstract

Many ultrafast phenomena in biology and physics are fundamental to our scientific understanding but have not yet been visualized owing to the extreme speed and sensitivity requirements in imaging modalities. Two examples are the propagation of passive current flows through myelinated axons and electromagnetic pulses through dielectrics, which are both key to information processing in living organisms and electronic devices. Here, we demonstrate differentially enhanced compressed ultrafast photography (Diff-CUP) to directly visualize propagations of passive current flows at approximately 100 m/s along internodes, i.e., continuous myelinated axons between nodes of Ranvier, from *Xenopus laevis* sciatic nerves and of electromagnetic pulses at approximately 5 × 10^7^ m/s through lithium niobate. The spatiotemporal dynamics of both propagation processes are consistent with the results from computational models, demonstrating that Diff-CUP can span these two extreme timescales while maintaining high phase sensitivity. With its ultrahigh speed (picosecond resolution), high sensitivity, and noninvasiveness, Diff-CUP provides a powerful tool for investigating ultrafast biological and physical phenomena.

## Abstract (web summary)

The authors introduce differentially enhanced compressed ultrafast photography, a phase imaging platform that combines high speed and sensitivity. They visualise propagation of passive current flows along myelinated axons, and electromagnetic pulses in lithium niobate.

## Introduction

Visualization of the spatiotemporal dynamics of propagation are fundamental to understanding dynamic processes in different areas of science and technology ranging from action potentials (APs) to electromagnetic pulses (EMPs), the two ultrafast processes in biology and physics, respectively^1,2^. AP propagation requires the coordinated action of two forms of current flow—passive flow along axons and active flow through voltage-dependent ion channels^3^. The AP conduction speed in both unmyelinated and myelinated axons is mainly limited by the opening of active ion channels; however, the speed in myelinated axons is accelerated by passive current flows, as the myelin sheath between two consecutive nodes of Ranvier is enclosed without active ion channels. Consequently, the conduction speed of APs ranges from 0.1 m/s in unmyelinated axons to 100 m/s in myelinated ones^1,4,5^. Recently, the development of advanced label-free phase imaging techniques^6,7^ enabled direct observation of APs propagating in unmyelinated axons or neurons^8–13^. However, it is often necessary to sacrifice imaging speed to achieve sufficient sensitivity to capture small phase changes resulting from AP firing. Concurrently, the direct visualization of fast APs propagating along internodes remains infeasible due to propagation over a short internode length^14,15^ (e.g., 100–300 µm) at high speed^5^ (e.g., 15–90 m/s). This dynamic phase event takes several microseconds to propagate through a micrometer-scale internode; hence, it is too fast to be captured using existing phase imaging techniques relying primarily on binning temporal frames to improve sensitivity^11,16,17^. Unlike APs, EMPs can propagate near the speed of light depending on the dielectric media^2^. Although propagating EMPs have been visualized indirectly using heterodyne schemes, pump-probe approaches, or ultrafast electron microscopy^18–20^, and the transverse profiles of propagating EMPs have been observed using an electro-optical sampling technique with a sub-100 fs temporal resolution^21^, complete direct observation of EMP propagation has not been achieved with existing methods due to insufficient sensitivity, low imaging speed, or low sequence depth (i.e., the total number of frames recorded in one shot). To directly observe these spatiotemporal dynamics of propagations, the imaging technique must have an ultrahigh speed, large sequence depth, and high sensitivity simultaneously, which cannot be achieved with existing techniques. We have previously reported compressed ultrafast photography (CUP), the world’s fastest camera that achieves both an ultrafast imaging of up to 70 trillion frames per second (fps) and a large sequence depth of up to 1000 frames^22–26^. We have also combined CUP with dark-field imaging and used pump and probe pulses to image ultrafast phase events in transparent objects^27^. However, without sufficient phase sensitivity (e.g., 0.9 mrad required to measure APs in spiking human embryonic kidney 293 (HEK-293) cells^11^), our previous CUP systems were not capable of imaging either propagating APs or weak EMPs.

To noninvasively, flexibly image the propagation of the fast transient excitation in optically transparent media, here we demonstrate differentially enhanced CUP (Diff-CUP), a phase imaging platform that integrates the ultrahigh speed and large sequence depth of CUP and the high sensitivity of a Mach–Zehnder interferometer^28^. Combined with a differential approach and a suitable field of view (FOV), Diff-CUP achieves a phase sensitivity of 20 µrad, the greatest phase sensitivity reported to date by an ultrafast imaging approach (approximately 0.12–0.3 mrad^11,13^). With a frame rate of up to 200 billion fps, a sequence depth of 350 frames, and the high phase sensitivity, Diff-CUP achieves noninvasive, direct spatiotemporal observation of passive internodal flows of electrical current, which plays a central role in AP propagation, and transmission of any other forms of electrical signaling across internodes where ion channel activities are absent^3^. Simulations of the spatiotemporal dynamics of propagating internodal current flows using a double cable circuit model^29^ are consistent with the experiment. The average conduction speed of the internodal current flows was measured to be approximately 100 m/s, which agrees well with the AP conduction speed in myelinated axons given by electrophysiological techniques^3,5^. We also visualized the fast propagating and evolving EMPs in lithium niobate (LN) based on the electro-optic effect. The measured conduction speed of approximately 5 × 10^7^ m/s agrees with the reported values^30,31^. Thereby, Diff-CUP enables imaging-based measurements of the highest speeds of propagating internodal current flows and EMPs and provides a potentially powerful tool for quantifying the dynamic processes in biology and physics.

## Results

### Diff-CUP system

**Fig. 1:**
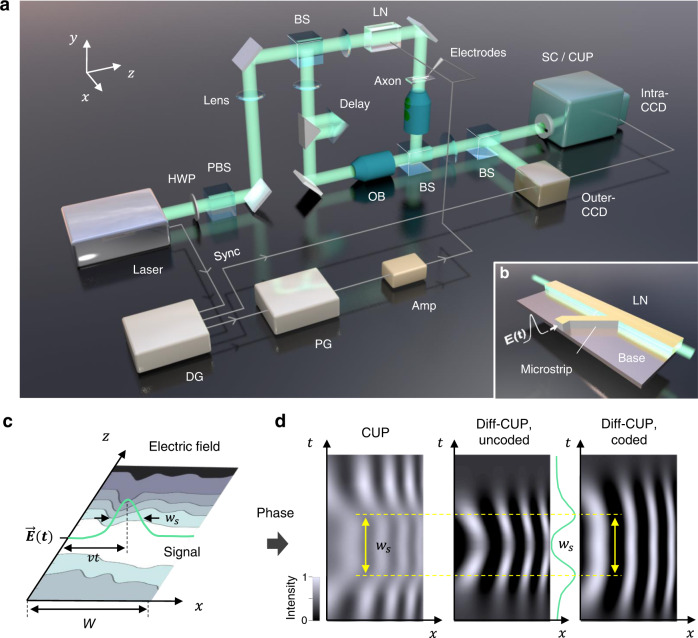
Diff-CUP system. **a** Schematic of the Diff-CUP system. Amp amplifier, BS beamsplitter, CCD charge-coupled device camera, DG delay generator, HWP half-wave plate, LN lithium niobate crystal, OB objective lens, PBS polarizing beamsplitter, PG pulse generator, SC streak camera, Sync synchronization. **b** Delivery of an EMP, $E\left(t\right)$, to the LN crystal through a custom-designed microstrip transmission line. **c** Spatiotemporal electric field distribution of the signal propagating in the LN crystal simulated using a finite element model. Green curve represents the Gaussian-shaped pulse in a snapshot. $W$, width of the LN crystal; $v$, propagation speed of the signal; ${w}_{s}$, pulse width of the signal. **d** Spatiotemporal phase changes resulted from the propagating signal in the LN crystal operating in different modes (conventional CUP, uncoded or coded). Yellow lines indicate the pulse width, and green lines represent the temporal intensity in the middle of the FOV.

The Diff-CUP system (Fig. 1a) consists of a Mach–Zehnder interferometer and a differentially enhanced lossless-encoding CUP detection system (Methods)^23,32^. Enabled by the high phase sensitivity of the interferometer^28^, a slight difference between the optical path lengths of the sample and reference arms leads to phase contrast images that can be detected by the CUP system. To realize synchronization of electrical and optical signals and observe sub-light propagation of EMPs, we installed an LN crystal in the sample arm of the interferometer. We designed a microstrip transmission line to maintain the pulse shape of the EMP when delivered to the LN crystal (Fig. 1b). The synchronization of the system can be found in Supplementary Fig. 1. The propagating EMPs generated transient changes of the electric field distribution within the LN crystal (Fig. 1c), which resulted in a refractive index change (birefringence) due to the linear electro-optic effect (Pockels effect). This created spatiotemporal optical path length differences between the sample and reference arms of the interferometer and, consequently, transient interferograms^33^. By placing myelinated axons prepared on a glass slide within the focal region of the objective lens in the sample arm, the Diff-CUP system can also be used to observe the fast propagation of internodal current flows in myelinated axons. Due to the spike-induced cellular deformations, propagating internodal current flows accompanying APs induce changes in the optical path length^8,11,13,34,35^, thereby also resulting in transient interferograms.

Time-unsheared and time-sheared views of the spatiotemporal interferograms are captured using an external charge-coupled device (CCD) and a fully opened streak camera with an internal CCD, respectively. Following the conventional CUP approach, the time-sheared views were spatially encoded with complementary binary patterns and divided into two distinct projections of the event. The time-unsheared view providing the original image information and time-sheared views providing lossless ultrafast process information were then used to reconstruct ultrafast phase events of up to 350 frames at 200 billion fps (see Methods). However, conventional CUP, even combined with a dark-field microscope and utilized a pump and a probe pulses to achieve a sensitivity of 3 mrad^27^, was not sufficiently sensitive to reconstruct the weak phase changes induced by events such as APs (e.g., 0.9 mrad in spiking HEK-293 cells^11^). In this work, we utilized a differential approach to trigger the dynamic phase events (APs and EMPs) in every other acquired interferogram. The resulting differential interferograms, i.e., the differences between the interferograms with and without dynamic phase events, were then processed to reconstruct phase images with a greater phase sensitivity. Meanwhile, we found that the phase sensitivity of Diff-CUP could be further improved by reducing the FOV using a slit to limit the spatial dimension along the time-sheared direction, and hence was high enough for extracting the weak signal in myelinated axons.

Considering the trade-off between phase sensitivity and FOV due to the combination of noises from different parts of the FOV that cannot be unmixed, we developed two operation modes in Diff-CUP. The first mode, termed the uncoded mode (with the slit narrowly opened), prioritized phase sensitivity over FOV. In uncoded mode, the captured images were differentiated (with and without signals), and then processed with averaging, temporal correlation, and template matching operations (Methods) to obtain the signal. The uncoded mode was capable of hypersensitive, ultrafast phase imaging to visualize signal propagation in narrow objects such as internodal current flows propagating in a myelinated axon (e.g., FOV of 2 mm × 0.05 mm). Movie frames could be recovered directly since the spatial and temporal information did not overlap. Additionally, the pulse shape of EMPs could be accurately extracted as a temporal correlation profile (see next section) through this mode. The second mode, termed the coded mode (with the slit fully opened), required an image reconstruction algorithm to distinguish the convolved spatial and temporal information in the differential interferogram. When operating in the coded mode, images were also differentiated and averaged before being decoded using the lossless-encoding CUP method^23,32^ (Methods). Then, a 2D correlation was performed between different frames to extract the signal envelope. This mode sacrificed phase sensitivity for FOV; nevertheless, enhanced by the proposed approaches, the coded mode still achieved a greater phase sensitivity than conventional CUP and enabled visualization of propagating EMPs over a large FOV (2 mm × 2 mm). A comparison among the time-sheared interferograms of a propagating EMP captured using conventional CUP and the two modes of Diff-CUP is shown in Fig. 1d. The pulse shape was “broadened” when the 2D spatial morphology was sheared, but it could be reconstructed by our CUP codes. The interferogram measured by Diff-CUP has a much higher visibility (modulation depth) compared to that acquired by conventional CUP, thus demonstrating the greater phase sensitivity of Diff-CUP.

### Hypersensitive phase imaging by Diff-CUP

**Fig. 2:**
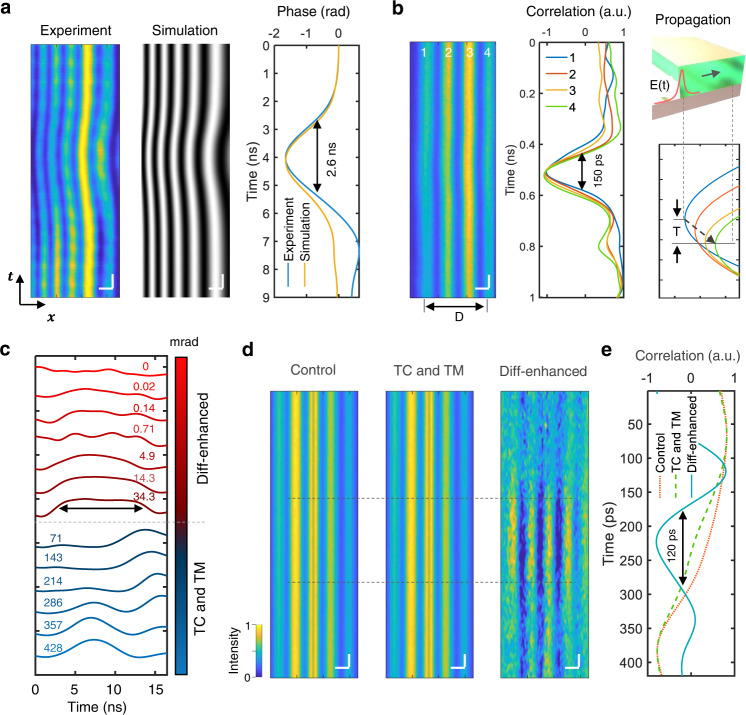
Phase sensitivity of Diff-CUP. **a** Spatiotemporal interferograms of a 2.6-ns EMP in the LN crystal captured by the streak camera and simulated by the electric field and phase distribution model, and the corresponding temporal phase averaging over the *x* direction. Horizontal scale bars, 300 µm. Vertical scale bars, 500 ps. **b** Experimental interferogram of a 150-ps propagating EMP and the corresponding temporal correlation of Fringes 1–4. D is the spatial distance between Fringes 1 and 4. The inset at the bottom right shows the approach to calculate the propagation time, T, of the EMP through the LN (top right). Horizontal scale bar, 300 µm. Vertical scale bar, 50 ps. **c** Quantification of phase-sensitivity of uncoded Diff-CUP by inducing EMPs with different amplitudes in the LN crystal. Each curve represents a reconstructed pulse shape with a different amplitude, where the corresponding phase change is shown above the curve. Black arrows denote the pulse width of the EMP. **d** Spatiotemporal interferograms of a weak EMP propagating in the LN crystal. Left, unprocessed interferogram, i.e., control group. Middle, interferogram processed with the temporal correlation (TC) and template matching (TM) methods. Right, interferogram processed with the TC, TM, and differential approach. Black dashed lines denote the regions of the EMP. Horizontal scale bars, 300 µm. Vertical scale bars, 20 ps. **e** Reconstruction of the pulse shape of the weak EMP using the three interferograms in (**d**).

Experimental and simulated (Supplementary Note 1) interferograms of the phase change caused by a 2.6-ns EMP are shown in Fig. 2a. Based on the good agreement between the measurements at high voltages and simulations using the electric field and phase distribution model (Supplementary Fig. 2), we deduced the phase change under the unresolved curvature of the interference fringes induced by weak EMPs from the simulation. The maximum phase change at a voltage of 116 V was estimated to be 1.6 rad. However, at lower voltages, phase changes less than 0.5 rad could not be discerned. Thus, we developed a correlation method for the uncoded mode, which was achieved by performing temporal correlation on the spatial rows and template matching on the correlation results (Methods). As shown in Supplementary Fig. 3, the temporal correlation combined with template matching method demonstrated superior performance over conventional denoising approaches including wavelet filtering^36^. Therefore, this method greatly improved the sensitivity. For instance, the weak phase change resulting from a 150-ps EMP was found in the correlation results (Fig. 2b). The EMP propagation can then be revealed by the temporal shifts between different fringes, T, and the propagation speed can be calculated as *v* = D/T, where D is the furthest spatial distance of the fringes. This procedure forms the basis of the propagation speed analysis throughout the paper. We then quantified the phase sensitivity of the method based on the electric field and phase distribution model and found that the greatest sensitivity reached 68 mrad (Fig. 2c). The phase sensitivity was obtained from the minimum stimulus that caused a raised correlation envelope distinguishable from the non-stimulus one. This moderate phase sensitivity, however, was insufficient to reconstruct the weak phase changes induced by current flows of APs or electromagnetic fields of EMPs, with small amplitudes. Moreover, this sensitivity was inferior to the 0.12–0.3 mrad phase sensitivity of existing imaging techniques employing frame-binning and lock-in detection^11,13^.

To improve the phase sensitivity, we applied temporal correlation and template matching to the differential interferograms between those with and without dynamic phase events (Supplementary Fig. 3). The differential approach combined with the temporal correlation and template matching methods enables the greatest phase sensitivity compared to that of the nondifferential approach or those not using temporal correlation or template matching (Supplementary Fig. 4). Therefore, the differential approach and the temporal correlation and template matching methods are both essential steps in ensuring the high sensitivity of Diff-CUP. As a result, while operating in the uncoded mode, Diff-CUP achieved a phase sensitivity of 20 µrad, which is the phase change caused by an EMP with an amplitude of 1 mV, the smallest voltage our system can detect (Supplementary Fig. 5). The 20 µrad phase sensitivity of Diff-CUP exhibits a 3400-fold enhancement over the 68 mrad phase sensitivity of the nondifferential approach (Fig. 2c and Supplementary Fig. 6). Whereas pump-probe schemes require frame binning to improve sensitivity—sacrificing temporal resolution, Diff-CUP does not because all the temporal information is contained in the spatiotemporal interferogram captured by the streak camera. The large difference in phase sensitivity between Diff-CUP and conventional approaches can be observed in Fig. 2d, where unprocessed, temporal correlation and template matching processed, and differentially enhanced interferograms of a weak EMP propagating in the LN crystal show clear distinctions both visually and quantitatively (Fig. 2e). For such a weak EMP signal, only the differential approach combined with temporal correlation and template matching in Diff-CUP can reconstruct the pulse. There are different types of noise in our system, including shot noise, the relative intensity noise of the laser, and the readout noise. These noises can be reduced by averaging the interferograms. However, this reduction is insufficient to reconstruct weak EMPs or internodal current flows accompanying APs (Supplementary Fig. 4). Another limit on sensitivity is the strong background in our interferograms caused by interference fringes, stray light, and optical distortions. This inhomogeneous background limits signal extraction because a tiny signal can be extracted from a near-zero or constant background (Supplementary Fig. 4g) rather than a varying background thousands of times stronger. The phase sensitivity of our system is determined by how the background and noises can be effectively suppressed below the signal level (Supplementary Figs. 6 and 10). As shown in Supplementary Fig. 6, the high phase sensitivity in imaging the weak EMP signal is enabled by the removal of the background and the sequential reduction of the noise through the Diff-CUP pipeline. In the final reconstruction result of Diff-CUP, the noise is suppressed to be lower than the EMP signal, thus enabling the faithful reconstruction of the pulse.

### Phase imaging of propagating internodal current flows in myelinated axons

**Fig. 3:**
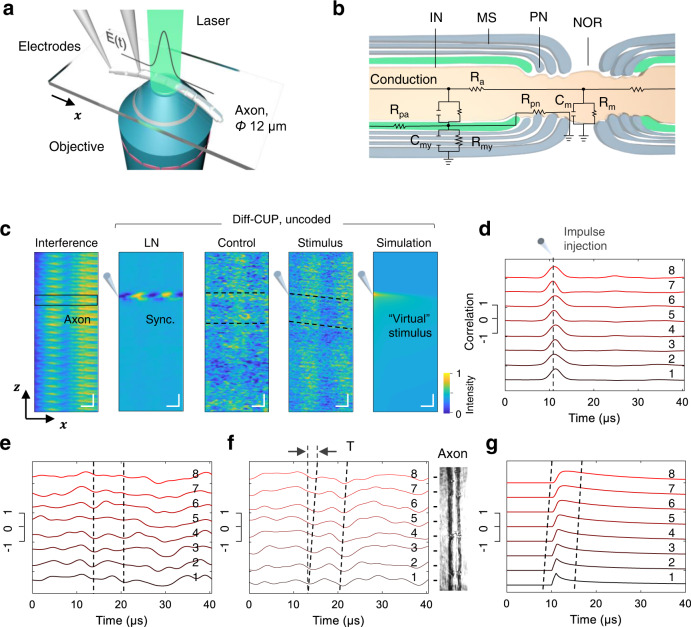
Uncoded Diff-CUP imaging of propagating internodal current flows in myelinated axons. **a** Setup for imaging propagating internodal current flows in the sample arm of Diff-CUP’s Mach–Zehnder interferometer. $E(t)$ is the transient field stimulation applied to the parallel bipolar microelectrodes. **b** Schematic of the equivalent double cable internodal circuit. IN internode, PN paranode, NOR node of Ranvier, MS myelin sheath; ${{{{{{\rm{R}}}}}}}_{{{{{{\rm{a}}}}}}}$, axonal axial resistivity; ${{{{{{\rm{R}}}}}}}_{{{{{{\rm{pa}}}}}}}$, periaxonal resistivity; ${{{{{{\rm{R}}}}}}}_{{{{{{\rm{pn}}}}}}}$, paranodal resistivity; ${{{{{{\rm{R}}}}}}}_{{{{{{\rm{m}}}}}}}$ and ${{{{{{\rm{C}}}}}}}_{{{{{{\rm{m}}}}}}}$, specific membrane resistance and capacitance; ${{{{{{\rm{R}}}}}}}_{{{{{{\rm{my}}}}}}}$ and ${{{{{{\rm{C}}}}}}}_{{{{{{\rm{my}}}}}}}$, specific myelin sheath resistance and capacitance. **c**, Spatiotemporal interferograms of a propagating internodal current flow in a myelinated axon captured by uncoded Diff-CUP (400 interferograms) under different conditions. The horizontal and vertical axes of the interferogram denote $x$ and $z$, respectively, where $z={nd}+y$, $x$ and $y$ are the two spatial axes, $n$ stands for the $n$-th axon image in the time series, and $d$ denotes the FOV in the $y$ dimension. From left to right: interference, unprocessed interferogram; LN, interferogram captured with a synchronous EMP induced in the LN crystal, where the strong Pockels effect caused by the EMP is used as a time reference for locating the starting time point of the current flow; control, interferogram captured without field stimulation; stimulus, interferogram captured with field stimulation; simulation, interferogram overlaid with a “virtual” stimulus generated by the NEURON simulation environment. Black box denotes the FOV of the axon. Gray electrode symbols denote when the stimulation is applied. Horizontal scale bars, 25 µm. Vertical scale bars, 3 µs. **d**–**g** Reconstructions based on the LN (**d**), control (**e**), stimulus (**f**), and simulation (**g**) interferograms in (**c**). Each reconstructed correlation curve corresponds to a segment of the FOV, labeled with numbers 1–8. Scale bars show the normalized correlation values. The regions corresponding to these segments are marked in the axon’s CCD image on the right of (**f**). Black dashed lines indicate the peak time of the synchronous EMP (**d**) or the signal region of the internodal current flow (**e**–**g**). T is the propagation time of the internodal current flow within the FOV of the axon.

Although AP spikes have been measured by various techniques^11,29^, observation of electrical current flows, which dominate AP propagation in neurons, remain elusive in research. Benefiting from the high phase sensitivity of uncoded Diff-CUP, we next imaged the fast propagation of internodal current flows in myelinated axons. Myelinated axons with a diameter of around 12 µm were teased out from the sciatic nerves of a *Xenopus laevis* frog and prepared on an adhesion microscope slide for Diff-CUP imaging (Fig. 3a, Methods). During the experiments, the axons were kept fully immersed in Ringer’s solution added onto the slide. Before imaging individual axons, we first evaluated the compound APs generated by the nerve bundle using a standard electrophysiological setup consisting of a nerve chamber and stimulating and recording electrodes (Supplementary Fig. 7). Based on the distance between electrodes and the time difference between the recorded stimulus and compound AP, we found the conduction speed of the compound APs to be around 31 m/s. By stimulating the axons with different pulse widths and peak voltages, we acquired the strength-duration curve of the axons^37–39^ (Supplementary Fig. 7). The 500-μs stimulus with a voltage threshold of 60 mV resulted in a 200-mV AP signal extracellularly with a gain of 50$\times$, whereas the 1-µs pulses, which had the shortest pulse width supported by our isolated pulse stimulator, had a stimulating voltage threshold of 8.5 V. We then used the 1-µs stimulating pulses with a peak voltage slightly higher than the threshold, i.e., 10 V, to inject passive internodal current flows in individual myelinated axons for Diff-CUP imaging. Compared to the broader pulses with lower stimulating thresholds (Supplementary Fig. 7), the passive current flows injected by 10-V, 1-µs pulses used here can induce larger cellular deformations and hence phase changes. Repeated reproduction of compound APs verified that the nerve was not damaged by the pulses (Supplementary Fig. 8). For Diff-CUP imaging, the 1-µs pulse and its propagation could be observed in a narrower (50 µs) time window with a 50-ns step, which is required to resolve the several-microsecond propagation through a micrometer-scale internode. We performed the phase imaging near the injected electrode before the induced signal was relayed and broadened by nodes of Ranvier that constrained by the all-or-none law. The differences between the internodal current flows observed by our Diff-CUP system and the APs observed through electrophysiology are illustrated in Supplementary Fig. 9.

To image an individual myelinated axon with the high phase sensitivity, it was necessary to tune the Diff-CUP system both spatially and temporally. Spatially, we positioned the prepared microscope slide such that the internode between nodes of Ranvier was placed at the center of the FOV (Fig. 3b). Temporally, we adjusted the time delay of the stimulating pulses to synchronize them with the imaging laser pulses and the time-shearing window of the streak camera. To indicate whether temporal synchronization was achieved and to locate the stimulus in the time axis, we routed a copy of the stimulator’s triggering signal to a pulse generator for inducing amplified EMPs in the LN crystal to induce an obvious dynamic phase event (Fig. 3c, d). To improve the signal-to-noise ratio (SNR) of Diff-CUP reconstruction, in each imaging session we captured 400 spatiotemporal interferograms with alternating stimulus and baseline, where stimulating pulses were sent to the field stimulation electrodes for every other interferogram. Since the phase changes of propagating internodal current flows could be very weak^8,11,13,34,35^ and the axon in the FOV might not survive the process, for every imaging dataset acquired with stimulation we also captured a dataset without stimulation as a control group (Fig. 3c, e). The imaging experiment was considered successful only if a propagating internodal current flow was observed in the dataset with stimulation but not in the control group (Fig. 3c, f). The experimental noise statistics in imaging axons shown in Supplementary Fig. 10 demonstrate the removal of the background and the sequential reduction of the noise through the Diff-CUP pipeline. The background and noise are suppressed to be lower than the internodal current flow signal in the Diff-CUP result, thus enabling reconstructing the signal above the noise floor. To corroborate the spatiotemporal profile of the propagating internodal current flow imaged by Diff-CUP, we simulated the same process with the NEURON simulation environment^40,41^ using the double cable model and the parameters described in Fig. 3b and Supplementary Note 2. The simulated current flow demonstrated a pulse width and conduction speed similar to the experimental result (Fig. 3c, g). As shown in Fig. 3f, the pulse width of the propagating internodal current flow in the myelinated axon is on the order of microseconds, while the long tail simulated in Fig. 3g could be too weak to be extracted in a noisy environment. In the reconstructed Diff-CUP movie (Supplementary Video 1), the dynamics of the propagating internodal current flow in the myelinated axon were consistent with the simulation. The discrepancies between the experiment and simulation were likely caused by the difficulty of including all the experimental conditions of Diff-CUP in the simulation.

To quantify the conduction speed imaged by Diff-CUP, we repeated the imaging experiment under the same conditions using six myelinated axons extracted from four animals (Supplementary Fig. 11, Supplementary Video 2). The conduction speeds of internodal current flows propagating in myelinated axons were calculated from the six groups of imaging data reconstructed by Diff-CUP. The mean and the standard error of the conduction speeds were 100 m/s and 26 m/s, respectively. These speeds were measured before the current flow propagated across a node; otherwise, recharging at nodes (unmyelinated) would take milliseconds, and the speed would be much lower. Compared to the 31 m/s speed of the compound AP measured using the synchronized electrophysiology setup, the internodal current flows on individual myelinated axons propagated faster. The lower conduction speed of the compound AP was likely a result of unmyelinated axons within the nerve bundle^42^ and the cumulative time required to generate a pulse strong enough to be detected. Most of the conduction speeds (Supplementary Fig. 11c) calculated from the Diff-CUP imaging data agreed with the 15–90 m/s speed of APs in 2.5–16 μm myelinated fibers in frogs^5^, but some surpassed this value. Compared to the slow 27 mm/s speed in unmyelinated spiking HEK-293 cells acquired by interferometric imaging^11^ and the 1–7 m/s speed in myelinated L5 pyramidal neurons measured by fluorescence imaging^29^, the 100 m/s averaged speed we measured on myelinated axons with Diff-CUP is the fastest AP-related conduction speed reported using an imaging-based approach.

### Phase imaging of propagating EMPs in an LN crystal

**Fig. 4:**
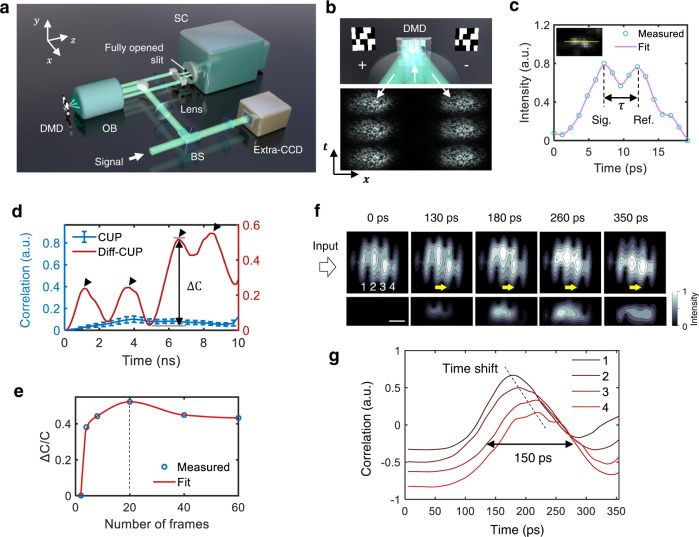
Coded Diff-CUP imaging of propagating EMPs in an LN crystal. **a** Lossless-encoding CUP setup in the Diff-CUP system. DMD, digital micromirror device; OB, objective lens; SC, streak camera; BS, beamsplitter. **b** Operation principle of lossless-encoding CUP. The DMD spatially encodes spatiotemporal scenes with a pseudorandom binary pattern by reflecting the incident light to either +12° (+) or –12° (–), creating two beams encoded with complementary patterns. **c** Quantification of temporal resolution in Diff-CUP. An 800-nm, 50-fs laser pulse (Libra-HE, Coherent) is split by the Mach–Zehnder interferometer into a reference pulse and a signal pulse. The temporal resolution of the system is measured as the minimum, distinguishable temporal distance between the two pulses with a 6 dB contrast-to-noise ratio. **d** Correlation between each frame and the first frame in the reconstructed movies of EMPs propagating in the LN crystal acquired by conventional CUP and coded Diff-CUP. The correlation curves indicate the phase change induced by the propagating EMPs. Data are presented as mean values ± standard errors of the means (*n* = 50). **e** Dependence of the maximum correlation change ($\triangle$C/C with C equals to one) in (**d**) on the number of interferograms used for coded Diff-CUP reconstruction. **f** Representative snapshots from the reconstructed movie of a 150-ps EMP (launched from left to right) propagating in the LN crystal acquired by coded Diff-CUP (20 interferograms). Shown below are the relative phase changes induced by the EMP obtained by subtracting the first frame from each frame in the movie. Numbers 1–4 denote the four interference fringes in the FOV. Yellow arrows indicate the propagating direction of the EMP. Scale bar, 500 µm. **g** Spatiotemporal profile of the propagating EMP revealed by processing the interference fringes 1–4 in (**f**) with the correlation and TM methods. Dashed line indicates the time shift of the EMP peaks. Black arrows denote the pulse width of the EMP.

Since the uncoded mode of Diff-CUP was specifically designed to image one-dimensional signal propagating in a transparent object, it was capable of hypersensitive phase imaging but had a long and narrow FOV, limiting the acquisition of spatial variation information along the temporal dimension. To image EMPs propagating and evolving in an LN crystal over a large FOV, we switched the Diff-CUP system to the coded mode, where the spatiotemporal interferograms created by the interferometer were directed to a lossless-encoding CUP setup before being imaged by the streak camera^23^ (Fig. 4a). Utilizing the complementary time-sheared views reflected from both the positive and negative pixels of the digital micromirror device (DMD), the coded Diff-CUP system prevented any loss of information from spatial encoding when reconstructing phase-sensitive images (Fig. 4b). In contrast to the conduction speed of internodal current flows in myelinated axons, the propagation speed of EMPs in an LN crystal is a sizeable fraction of the speed of light^30,31^. Therefore, it takes around tens of picoseconds for an EMP to propagate through the 2 mm $\times$ 2 mm FOV of coded Diff-CUP, a process that can be resolved by the 5 ps temporal resolution of Diff-CUP (Fig. 4c; note that greater temporal resolution can be simply obtained using a faster streak camera). To evaluate the phase sensitivity of coded Diff-CUP, we used both conventional CUP and coded Diff-CUP to reconstruct the movies of a train of EMPs propagating in an LN crystal (Supplementary Video 3). We then conducted a correlation analysis using each frame of the reconstructed movies to quantify the maximum correlation change, $\triangle {{{{{\rm{C}}}}}}/{{{{{\rm{C}}}}}}$ (the difference between the maximum and minimum correlation values, $\triangle {{{{{\rm{C}}}}}}$, normalized by the theoretical maximum correlation value, ${{{{{\rm{C}}}}}}=1$), and found that coded Diff-CUP exhibited a 338-fold enhancement over conventional CUP (Fig. 4d). By inducing EMPs with different amplitudes in the LN crystal, we further quantified the phase sensitivity of coded Diff-CUP to be around 3 mrad (Supplementary Fig. 12). Meanwhile, using $\triangle$C/C as a metric, we found the optimal number of interferograms used for coded Diff-CUP reconstruction to be 20 (Fig. 4e), much fewer than the 300–400 interferogram used in the uncoded mode. This distinction can be attributed to the degraded spatial encoding when averaging many interferograms^23^, and also results in the lower sensitivity of coded Diff-CUP compared to that of uncoded Diff-CUP.

To image the propagation and evolution of a single EMP while retaining optimal phase sensitivity, we captured 20 spatiotemporal interferograms for coded Diff-CUP reconstruction, where a 150-ps EMP was induced in the LN crystal in every other interferogram. In the reconstructed coded Diff-CUP movie (Fig. 4f, Supplementary Video 4), the two-dimensional phase change induced by the EMP propagated from left to right with a stutter at the interfaces between the microstrip and LN. Equally spaced across the FOV, the four interference fringes in Fig. 4f can be used to analyze the spatiotemporal dynamics of the EMP. We performed 2D correlation between adjacent frames and observed the propagation of the EMP as its peak shifted temporally (Fig. 4g). By linearly fitting the relation between the time shifts of the EMP peaks and the locations of the interference fringes, we calculated the propagation speed of the 150-ps EMP in the LN crystal to be about 5 × 10^7^ m/s. This speed corresponds to a relative permittivity of 36, agreeing with the reported relative permittivity of LN at high frequency, i.e., 25–45 ^30,31^. To corroborate the dynamics of the propagating EMP acquired by Diff-CUP, we also simulated the electrical field distribution within an LN crystal (Supplementary Fig. 13a) using an electric field and phase distribution model (Supplementary Note 1, Supplementary Video 5)^43^. The correlations calculated by CUP, coded Diff-CUP, and the model were consistent (Supplementary Figs. 13b, c). The experimental phase changes and simulated electrical field induced by the EMP are presented one-to-one as shown in Supplementary Fig. 13d, which may be used to study the ultrafast spatiotemporal dynamics in some special cases. Overall, unlike the visualization of electromagnetic waves propagating at a beating frequency implemented with heterodyne or pump-probe approaches^18–21^, our Diff-CUP system operating in the coded mode enabled the direct visualization of a weak EMP traveling at sub-light speed.

## Discussion

With a frame rate of up to 200 billion fps, a sequence depth of up to 350 frames, and a phase sensitivity of 20 µrad, Diff-CUP has enabled direct imaging of internodal current flows propagating in myelinated axons with an average conduction speed of 100 m/s as well as the direct visualization of an EMP propagating in an LN crystal with a speed of 5 × 10^7^ m/s. The reproduced internodal current flow speed and LN relative permittivity are consistent with the simulated and reported values, demonstrating the translation of our method into the biological and physical sciences. However, because of this high phase sensitivity, the differential approach used in both modes of Diff-CUP requires the dynamic phase events to be repeatable. To image non-repeatable ultrafast events, such as the optical Kerr effect in a crystal or laser-induced shock wave propagation in water, Diff-CUP can operate non-differentially (i.e., single shot, see Fig. 2a, b) with a lower phase sensitivity. At the same time, the differential method of our Diff-CUP system can be readily applied to our previous non-phase CUP systems^22–24^ to capture low-intensity light such as fluorescence excitation. Considering ultrafast imaging of different conditions can be obtained, Diff-CUP offers a platform that can be flexibly configured to prioritize one imaging property (sensitivity, speed, and FOV) over the others.

Because our phase imaging setup is based on a Mach–Zehnder interferometer and operated in transmission mode, the sample needs to be thin enough to not severely disrupt the interference pattern. This requirement limits the complexity of the neural structures that can be imaged. Constrained by the time-shearing window of the streak camera, our method currently could not image dynamic phase events lasting longer than 1 ms. Therefore, it is not suitable to image slow dynamics such as APs lasting for several milliseconds or their propagation in unmyelinated axons or within a neuronal network. However, this limitation can alternatively be solved by replacing the streak camera with one that has a longer time-shearing window, or using state-of-the-art high-speed time-sheared CCD or CMOS cameras^44–46^, sacrificing nanosecond or picosecond resolution^11^. Meanwhile, limited by the electrical bandwidth of the LN crystal (10 GHz) determined by parasitic capacitance, our crystal is not suitable to inject EMPs with a pulse width shorter than 100 ps. Even though the bandwidth of LN crystals can be increased to around 100 GHz using integrated optics^47,48^ with a physical width reduced to below 100 µm, they are often fiber-based and hence would have a strong scattering in interference imaging and a several-picosecond crossing time, which is beyond the temporal resolution of our streak camera. Another limitation of our method is the unreliability in extracting the actual phase values directly from the differential interferograms. Although the waveform amplitudes were supposed to yield accurate phase profiles for the temporal correlation and template matching methods, this method exhibits high uncertainty due to the high noise level and inhomogeneous background and is unable to quantify the tiny phase change of internodal current flows. As for differentiation, the phase change extracted by correlation was similar (Supplementary Figs. 5b–g). This approach can be explained by the equations given in Methods: the correlation of the first line, ${s}_{0}$, and the signal lines, $s$, in the differential interferogram is ${{{{{\rm{Corr}}}}}}\left[D\left(x,\;{y}_{0};{t}_{0}\right),\;D\left(x,\;{y}_{0};{t}_{s}\right)\right]={{{{{\rm{Corr}}}}}}\left[{s}_{0},\;s\right]$. When the signal amplitude changes by a constant factor $a$ by varying the excitation voltage, the correlation coefficient is unchanged because ${{{{{\rm{Corr}}}}}}\left[D\left(x,\;{y}_{0};{t}_{0}\right),\;D\left(x,\;{y}_{0};{t}_{s}\right)\right]={{{{{\rm{Corr}}}}}}\left[{{as}}_{0},\;{as}\right]={{{{{\rm{Corr}}}}}}\left[{s}_{0},\;s\right]$. Therefore, different voltages result in similar profiles of the phase change. It remains a significant challenge to accurately deduce the actual phase changes of internodal current flows because of the irregular interference fringes disturbed by the axon morphology and curved fluid surface, which compromise the sensitivity of our Diff-CUP system. Finally, complex or arbitrary spatiotemporal phase variation is more difficult to quantify using our method because the compressed sensing algorithm requires the object to be sparse in some space^24^. This restriction on sparsity may be alleviated by sacrificing the temporal resolution or optimizing the compressed sensing algorithm.

With the development of femtosecond lasers, ultrafast pump-probe methods that use ultrashort pulses to improve spatial and temporal resolution are rapidly evolving. However, the current main limitation for these approaches may be the common dependence on a relatively complex laser system that can generate two or more stable and well synchronized ultrashort (approximately tens to hundreds of femtosecond) pulse sequences^49^. A recently reported pump-probe scheme (stimulated Raman scattering) to visualize the puff-induced depolarization of multiple neurons merely achieved a line rate of 750 Hz^12^ and a frame rate of 10 Hz^50^. Moreover, pump-probe methods mostly use slow CCDs and Fourier transform for inverse transformation to indirectly obtain the final phase and amplitude image^51–53^, while our method used a nanosecond laser and a streak camera with variable temporal resolutions to directly acquire dynamic processes despite a convolution of 2D spatial and temporal information. Other label-free modalities for imaging of APs mainly include optical coherence tomography (1 kHz line rate on Aplysia neurons^16^), interferometric imaging (1 kHz frame rate on HEK293 cells^11^), transmission light (1.6 kHz frame rate on mammalian neurons^17^), and second harmonic generation (1.7 Hz frame rate on mammalian neurons^54^). These methods achieved much lower imaging speeds compared to the frame rate of Diff-CUP, which does not compromise frame depth and sensitivity.

In summary, enabled by the ultrahigh imaging speed, large sequence depth, and high phase sensitivity, Diff-CUP allows observation of fast internodal current flow propagations, which has not yet been realized, in myelinated axons, as well as sub-light EMP spatiotemporal evolutions in an LN crystal. These are among the fastest biological and physical signals that are rarely investigated. Particularly, the differential approach utilizing alternative stimulations can be immediately applied in other imaging modalities calling for high imaging sensitivity. We envision that, with the capacity to image important biological and physical processes that have not been directly observed before, Diff-CUP opens the door for investigation of more biological and physical phenomena that are too fast or too weak to be observed using conventional imaging approaches. It can span broad timescales while maintaining high phase sensitivity. Therefore, the technique could find applications in investigating ultrafast biological and physical phenomena, such as neural activities^6^, catalytic processes in enzymes^55^, and sonoluminescence^56^.

## Methods

### Diff-CUP system and principle

In the Diff-CUP system (Fig. 1a), the power of a nanosecond pulsed laser beam at 532 nm (VGEN-G-10, Spectra-Physics) was controlled by a half-wave plate (WPH10M-532, Thorlabs) and a polarizing beamsplitter (PBS251, Thorlabs). The laser beam was then reflected by a mirror, focused through a lens (AC508-150-A, Thorlabs), and split into two arms of a Mach–Zehnder interferometer by a beamsplitter (BS013, Thorlabs). Both arms consisted of a lens (AC254-050-A, Thorlabs), a mirror, and an objective lens (M-10X, Newport; 10$\times$,0.25NA). In the sample arm, the beam passed through an LN crystal (4001NF, New Focus; 40 mm $\times$ 4 mm $\times$ 2 mm) before entering the objective lens, and a glass slide (shown in Fig. 3a) on an XYZ-stage (MT-XYZ, Newport) was used to place the teased axons within the focal region of the objective lens. The time-averaged power of the laser at the sample was about 1 mW, corresponding to a radiant exposure (incident fluence) of approximately 2 µJ/cm^2^, which was far below the American National Standards Institute safety limit^57^ (20 mJ/cm^2^ at 532 nm). The laser had a pulse width of 20 ns and a pulse repetition rate of 700 kHz; therefore, the axon was exposed to multiple laser pulses during the experiment, repeating images of the axons in the spatiotemporal interferograms (as shown in Fig. 3c and Supplementary Figs. 10, 11). In the reference arm, a knife-edge right-angle prism mirror (MRAK25-P01, Thorlabs) and a right-angle prism (PS911, Thorlabs) were used to compensate for the optical path length difference between the two arms. The beams in the two arms were recombined using another beamsplitter (BS013, Thorlabs) and passed through the tube lens (AC508-180-A, Thorlabs) of the objectives before being directed to the streak camera (C7700, Hamamatsu) in a lossless-encoding CUP setup^23,32^ (shown in Fig. 4a). A beamsplitter (BS013, Thorlabs) was placed before the lossless-encoding CUP setup to reflect a part of the light to an external CCD camera (FMVU-03MTM-CS, FLIR).

The operation mode of Diff-CUP was determined by the DMD (DLP3000, Texas Instruments) and the slit in the lossless-encoding CUP setup. In the uncoded mode, the DMD was loaded with a blank pattern and the slit was slightly opened, hence creating a single spatiotemporal integration, $E\left(x,\;{y}_{0}{;t}\right)$, where ${y}_{0}$ was limited to a small FOV due to the slightly opened slit. In the coded mode, on the other hand, the DMD was loaded with a pseudorandom binary pattern and the slit was fully opened, thus creating two spatiotemporal integrations coded with complementary patterns, $E\left(x,\;{y;t}\right)$ and ${E}^{{\prime} }\left(x,\;{y;t}\right)$ (Fig. 4b), where $y$ corresponded to a larger FOV captured with the fully opened slit. A delay generator (DG645, Stanford Research Systems) was triggered by the laser pulse signal detected by a photodiode (DET10A, Thorlabs). The triggered signal was downscaled by a delay generator to provide trigger inputs for the external CCD camera, the streak camera, a pulse generator used to induce EMPs in the LN crystal, and a stimulator used to inject passive current flows in the axons, thus enabling synchronization among all the instruments. In a Diff-CUP imaging experiment, the trigger rates for the external CCD and streak cameras were 50 Hz, while the rates for the pulse generator and the stimulator were 25 Hz. Therefore, within the raw CCD and streak camera images captured at a 50 Hz frame rate, the signals, i.e., the EMPs and the internodal current flows, were presented in the raw images in every other interferogram. Since EMPs and internodal current flows were repeatable events, we acquired multiple raw interferograms, $E$, in a Diff-CUP experiment, and we took the difference between the interferograms with and without signals, i.e., $D={E}_{{{{{{\rm{signal}}}}}}}-{E}_{{{{{{\rm{nosignal}}}}}}}$. The resulting differential interferograms of the uncoded Diff-CUP, $D\left(x,\;{y}_{0}{;t}\right)$, were processed to create hypersensitive phase movies with a small FOV. On the other hand, the differential interferograms of the coded Diff-CUP, $D\left(x,\;{y;t}\right)$ and ${D}^{{\prime} }\left(x,\;{y;t}\right)$, were processed to generate ultrafast phase movies with a large FOV. Note that the differential approach here is essentially a form of software lock-in detection.

### Preparation of myelinated axons and injection of internodal current flows

The single myelinated axons were dissected from a female 9+ cm *Xenopus laevis* (LM00535, Nasco) following the procedures as described^58–60^. All the laboratory animal protocols were approved by the Institutional Animal Care and Use Committee of the California Institute of Technology. Before dissection, the *Xenopus laevis* was euthanized by immersion in >0.5 g/L tricaine methanesulfonate (MS222) buffered with sodium bicarbonate for a pH between 7 and 7.5 for an hour. Excision of the heart was then used to ensure death. After euthanasia, blunt-tip scissors (14084-09, FST) and curved forceps (11051-10, FST) were used to cut the skin to expose the biceps femoris and the intermuscular septum. Spring scissors (15010-10, FST) and fine forceps (Dumont #5, FST) were used to separate the biceps femoris along the intermuscular septum to expose the sciatic nerve. A nerve bundle was excised by the spring scissors from the sciatic nerve and stored in a petri dish filled with Ringer’s solution (LRE-S-LSG-1004-3, Ecocyte Bioscience). To confirm whether the nerve bundle survived the procedure, it was placed over the stainless-steel wire electrodes inside a nerve chamber (MLT016, ADInstruments) filled with Ringer’s solution, where the two pairs of electrodes were connected to an isolated pulse stimulator (Model 2100, A-M Systems) for stimulating and a differential amplifier (Model 3000, A-M Systems) for recording, respectively. The output of the differential amplifier was then read by a custom-written LabVIEW (National Instruments) program through a data acquisition card (ATS9350, AlazarTech) installed on a desktop computer.

After acquiring successful electrical recordings of the nerve bundle’s compound APs in response to stimulations, the nerve bundle was transferred to an adhesion microscope slide (48382-117, VWR), and the individual myelinated axons were teased out using the fine forceps and spring scissors under a phase-contrast microscope (TS100, Nikon). During the procedure, more Ringer’s solution was added onto the dissection slide to keep the axons immersed in the solution. The dissection slide with the teased axons was then transferred to the Diff-CUP system for imaging. The slide was placed on the XYZ-stage above the objective lens in the sample arm. After a myelinated axon with visible nodes of Ranvier was located within the FOV, a pair of parallel bipolar microelectrodes (30211, FHC), controlled by a micromanipulator system (MPC-200, Sutter Instrument), was placed near the axon to create field stimulation. The position of the electrodes was carefully adjusted such that the electrode tips were immersed in Ringer’s solution but not in direct contact with the axon, and the fringes in the interferogram were as wide as possible. To inject passive current flows in myelinated axons by field stimulation, the isolated pulse stimulator was used to deliver stimulating pulses with different amplitudes and pulse widths to the electrodes. The stimulator was triggered by a function generator (33250 A, Agilent), which was synchronized with the delay generator. If properly configured, the stimulator would inject passive current flows in the axons in every other interferogram during a Diff-CUP imaging experiment. However, despite the patient and meticulous operations, we had a small success rate of <40% in obtaining the internodal current flow propagation using Diff-CUP throughout the whole research. This could be attributed to dissection failures, accidental contacts with metal, unstable transfer, or excessive stimulations (Supplementary Note 3).

### Induction of EMPs in an LN crystal

The EMPs propagating in the LN crystal imaged by the Diff-CUP system were induced by an ultra-wideband pulse generator (GZ1117DN-20, Geozondas; 30 ps pulse width). To induce picosecond EMPs with different amplitudes, 30-ps pulses were passed through different configurations of a monolithic amplifier (PHA-102+, Mini-Circuits; 0.05–6 GHz bandwidth), a power amplifier (HMC8205BF10, Analog Devices; 0.3–6 GHz bandwidth), and attenuators (FW-X+, Mini-Circuits) with various attenuation powers before being directed to the LN crystal. Due to the lower bandwidth of the amplifiers compared to that of the pulse generator, the resulting pulse width of the EMPs delivered to the LN crystal was around 150 ps. All the components in the radiofrequency (RF) pathway were matched at 50 Ω and connected using 40 GHz precision test cables with 2.92 mm connectors. To avoid signal distortion and reflection due to electrical impedance mismatch, a custom-designed microstrip transmission line (5 × 50 × 1.575 mm^3^ dimension, Rogers RT/duroid 5880 substrate, 49.27 Ω) was fabricated to deliver the EMPs to the LN crystal. The microstrip line and the LN crystal were connected via a 135° mitered bend to further reduce the reflection of the EMPs from the boundary^61^ (shown in Fig. 1b). The pulse generator, triggered by the delay generator, would induce EMPs in the LN crystal in alternating interferograms during a Diff-CUP imaging experiment.

### Data processing and image reconstruction

When operating in the uncoded mode, the differential interferograms $D\left(x,\;{y}_{0}{;t}\right)$ were averaged and processed with the correlation and template matching methods, resulting in hypersensitive phase imaging. The correlation method measured the cross-correlation between the first line $D\left(x,\;{y}_{0};{t}_{0}\right)$ with the other lines in the interferogram, i.e., $C\left(x,\;{y}_{0}{;t}\right)={{{{{\rm{Corr}}}}}}\left[D\left(x,\;{y}_{0}{;t}\right),\;D\left(x,\;{y}_{0};{t}_{0}\right)\right]$. We used the first line as the reference because it contained the signal at the beginning of propagation (we used the delay generator to ensure that the signal was located at the center of the time window). Without the differential operation, the first line in the interferogram can be written as $E\left(x,\;{y}_{0};{t}_{0}\right)={s}_{0}+b+{n}_{0}$, where ${s}_{0}$ denotes the signal at ${t}_{0}$, $b$ is the background, and ${n}_{0}$ denotes the shot noise in the first line. A signal line can be written as $E\left(x,\;{y}_{0};{t}_{s}\right)=s+b+{n}_{s}$, where $s$ is the signal at ${t}_{s}$, and ${n}_{s}$ is the shot noise in the signal line. Their correlation, therefore, can be written as ${{{{{\rm{Corr}}}}}}\left[E\left(x,\;{y}_{0};{t}_{0}\right),\;E\left(x,\;{y}_{0};{t}_{s}\right)\right]={{{{{\rm{Corr}}}}}}\left[{s}_{0},\;s\right]+{{{{{\rm{Corr}}}}}}\left[{s}_{0}+s,b\right]+{{{{{\rm{Corr}}}}}}\left[b,\;b\right]$, where the shot noise ${n}_{0}$ and ${n}_{s}$ are uncorrelated. With the differential operation, the background $b$ is eliminated. Therefore, the first line in the differential interferogram can be written as $D\left(x,\;{y}_{0};{t}_{0}\right)={{s}_{0}+n}_{0}^{{\prime} }$, where ${n}_{0}^{{\prime} }$ denotes the shot noise in the differentiated first line. The signal line, on the other hand, can be written as $D\left(x,\;{y}_{0};{t}_{s}\right)={s+n}_{s}^{{\prime} }$, where ${n}_{s}^{{\prime} }$ is the shot noise in the differentiated signal line. Hence, their correlation is simply ${{{{{\rm{Corr}}}}}}\left[D\left(x,\;{y}_{0};{t}_{0}\right),D\left(x,\;{y}_{0};{t}_{s}\right)\right]={{{{{\rm{Corr}}}}}}\left[{s}_{0},\;s\right]$, where ${n}_{0}^{{\prime} }$ and ${n}_{s}^{{\prime} }$ are also uncorrelated. The advantage of the differential operation can be observed by comparing ${{{{{\rm{Corr}}}}}}\left[E\left(x,\;{y}_{0};{t}_{0}\right),\;E\left(x,\;{y}_{0};{t}_{s}\right)\right]$ and ${{{{{\rm{Corr}}}}}}\left[D\left(x,\;{y}_{0};{t}_{0}\right),\;D\left(x,\;{y}_{0};{t}_{s}\right)\right]$. While the former has background-related terms, i.e., ${{{{{\rm{Corr}}}}}}\left[{s}_{0}+s,\;b\right]$ and ${{{{{\rm{Corr}}}}}}\left[b,\;b\right]$, which prevent the extraction of the signal, the latter is clean with only a single term ${{{{{\rm{Corr}}}}}}\left[{s}_{0},\;s\right]$, which is related to the signal without the background term. Therefore, the differential and temporal correlation method here turns out to be an effective approach to extract the weak signal from the background. The template matching method then cross-correlated $C\left(x,\;{y}_{0}{;t}\right)$ with a Gaussian function, i.e., ${C}_{{{{{{\rm{TM}}}}}}}\left(x,\;{y}_{0}{;t}\right)={{{{{\rm{Corr}}}}}}[C\left(x,\;{y}_{0}{;t}\right),\;G(t,\;{w}_{g})]$, where $G(t,\;{w}_{g})$ was a Gaussian template with a full width at half maximum (FWHM) of ${w}_{g}$. ${w}_{g}$ was set according to the pulse width of the EMPs and internodal current flows experimentally measured with an oscilloscope or a data acquisition card. The internodal current flow propagation movies (Supplementary Videos 1 and 2) were obtained by merging the time shift information into the CCD image of the axons. When operating in the coded mode, the differential interferograms $D\left(x,\;{y;t}\right)$ and ${D}^{{\prime} }\left(x,\;{y;t}\right)$ were averaged and processed following the lossless-encoding CUP method described in Refs. 23, 32. Briefly, the measured intensity distributions of the streak camera and the external CCD, $I$, is related to the photon distribution of the dynamic scene $P(x,\;{y},\;{t})$ as $I={{{{{\boldsymbol{O}}}}}}P$, where $I={[{I}^{(0)},\;{a}{I}^{(1)},\;{a}{I}^{(2)}]}^{T}$ and ${{{{{\boldsymbol{O}}}}}}={[{{{{{\boldsymbol{T}}}}}}{{{{{{\boldsymbol{L}}}}}}}_{0},\;{a}{{{{{\boldsymbol{TS}}}}}}{{{{{{\boldsymbol{K}}}}}}}_{1}{{{{{{\boldsymbol{L}}}}}}}_{1}{{{{{{\boldsymbol{M}}}}}}}_{1},\;{a}{{{{{\boldsymbol{TS}}}}}}{{{{{{\boldsymbol{K}}}}}}}_{2}{{{{{{\boldsymbol{L}}}}}}}_{2}{{{{{{\boldsymbol{M}}}}}}}_{2}]}^{T}$. The linear operator ${{{{{\boldsymbol{T}}}}}}$ represents the spatiotemporal integration, ${{{{{{\boldsymbol{L}}}}}}}_{j}$ ($j=0,\;1,\;2$) represents the spatial low-pass filtering due to optics, ${{{{{\boldsymbol{S}}}}}}$ represents the temporal shearing, ${{{{{{\boldsymbol{K}}}}}}}_{i}$ ($i=1,\;2$) represents the image distortion primarily owing to the encoding arm, and ${{{{{{\boldsymbol{M}}}}}}}_{i}$ ($i=1,\;2$) represents the complementary spatial encoding masks with ${{{{{{\boldsymbol{M}}}}}}}_{1}+{{{{{{\boldsymbol{M}}}}}}}_{2}=1$. The scalar factor $a$ is related to the energy calibration of the streak camera against the external CCD camera. Using the complementary differential interferograms, ${I}^{(1)}=D\left(x,\;{y;t}\right)$ and ${I}^{(2)}={D}^{{\prime} }\left(x,\;{y;t}\right)$, the time-unsheared image captured by the external CCD, ${I}^{(0)}$, and the known operator ${{{{{\boldsymbol{O}}}}}}$ as inputs, the ultrafast picosecond-resolution phase images with space and intensity constraints were recovered utilizing the two-step iterative shrinkage/thresholding algorithm^62^.

### Computational modeling of propagating internodal current flows and EMPs

Computational modeling of propagating internodal current flows in myelinated axons was performed with the NEURON simulation environment (v7.7)^40,41^, which has been widely used in laboratories and classrooms around the world due to its accuracy in modeling the neuronal dynamics observed experimentally. Following the double cable model and the optimal cable parameters described in Supplementary Note 2^63,64^, a custom-written Python model was developed to simulate the nodal, internodal, and paranodal domains in a myelinated axon and the saltatory conduction along the axon. Computational modeling of the EMP’s propagation in the microstrip line and the LN crystal was performed using a finite element modeling software (COMSOL Multiphysics). A 3D computer-aided design model was created based on the physical dimensions of the microstrip line and the LN crystal (Supplementary Video 5). Materials properties were assigned based on the values reported in the literature (LN^30^) or provided by the vendor (Rogers RT/duroid 5880 substrate). The EMP was defined in the model by setting the boundary condition of the lumped SMA port of the microstrip line according to the picosecond pulse shape measured experimentally using an oscilloscope. Modeling results were exported as transient 3D volumetric movies of the electric field distribution.

### Reporting summary

Further information on research design is available in the Nature Research Reporting Summary linked to this article.

## Supplementary information

Supplementary Information

Description of Additional Supplementary Files

Supplementary Video 1

Supplementary Video 2

Supplementary Video 3

Supplementary Video 4

Supplementary Video 5

Reporting Summary

## Data Availability

All data are available within the Article and Supplementary Information, or available from the corresponding author upon reasonable request.

## Code availability

All custom codes used in this study are available from the corresponding author upon reasonable request.
