# Supplementary material for: Ultrafast and hypersensitive phase imaging of propagating internodal current flows in myelinated axons and electromagnetic pulses in dielectrics
Source: Nat Commun. 2022 Sep 6;13:5247. doi: 10.1038/s41467-022-33002-8 (PMC9448739; doi:10.1038/s41467-022-33002-8)
Supplement: Supplementary file 1 — Supplementary Information [file 41467_2022_33002_MOESM1_ESM.pdf]

Supplementary Information

Ultrafast and hypersensitive phase imaging of propagating internodal current flows in myelinated axons and electromagnetic pulses in dielectrics

Yide Zhang[1†], Binglin Shen[1,2†], Tong Wu[1,3], Jerry Zhao[1], Joseph C. Jing[1], Peng Wang[1], Kanomi Sasaki-Capela[4], William G. Dunphy[4], David Garrett[1], Konstantin Maslov[1], Weiwei Wang[5], and Lihong V. Wang[1*]

[1]Caltech Optical Imaging Laboratory, Andrew and Peggy Cherng Department of Medical Engineering, Department of Electrical Engineering, California Institute of Technology, Pasadena, CA 91125, USA

[2]Present address: Key Laboratory of Optoelectronic Devices and Systems of Guangdong Province and Ministry of Education, College of Physics and Optoelectronic Engineering, Shenzhen University, Shenzhen 518060, China

[3]Present address: Key Laboratory of Space Photoelectric Detection and Perception, Nanjing University of Aeronautics and Astronautics, Nanjing 210016, China

[4]Division of Biology and Biological Engineering, California Institute of Technology, Pasadena, CA 91125, USA

[5]Department of Biophysics, University of Texas Southwestern Medical Center, Dallas, TX 75390, USA

† These authors contributed equally.

* Correspondence should be addressed to L.V.W. (LVW@caltech.edu).

Supplementary Note 1 Electric field and phase distribution model

The electromagnetic (EM) wave in the frequency domain can be simulated using the time-domain form of Maxwell's equations,

$$\nabla \times \mu_r^{-1}(\nabla \times \mathbf{A}) + \mu_0 \sigma \frac{\partial \mathbf{A}}{\partial t} + \mu_0 \frac{\partial}{\partial t}\left(\varepsilon_0 \varepsilon_r \frac{\partial \mathbf{A}}{\partial t}\right) = 0, \tag{S1}$$

where μ_r and μ_0 are the relative permeability and vacuum permeability, respectively; ε_r and ε_0 are the relative permittivity and vacuum permittivity, respectively; σ is the electrical conductivity. **A** is the magnetic vector potential defined by

$$\begin{aligned} \mathbf{B} &= \nabla \times \mathbf{A}, \\ \mathbf{E} &= -\nabla \phi - \frac{\partial \mathbf{A}}{\partial t}, \end{aligned} \tag{S2}$$

where **B** is the magnetic field, **E** is the electric field, and ϕ is the electric potential (a scalar field). We used the perfect electric conductor (PEC),

$$\mathbf{n} \times \mathbf{E} = 0, \tag{S3}$$

as the boundary condition that sets the tangential component of the electric field to zero. This is reasonable because the losses in the dielectric substrate usually far exceed the losses at any metallization in microwave circuits.

The resulting electric field in the y direction, E_y (y is the axis perpendicular to the electrodes), can be used to calculate the time-dependent phase change due to the Pockels effect, $\Delta\varphi(x, y, z, t)$, using the finite element method (FEM) approach:

$$\Delta\varphi = \frac{\pi \delta y}{V_\pi} E_y + \varphi_0, \tag{S4}$$

where V_π is the half-wave voltage of the Pockels effect given by

$$V_\pi = \frac{\lambda \delta y}{2n^3 r_{22} \delta z}, \tag{S5}$$

where λ is the optical wavelength, n is the refractive index of the LN crystal, and r_{22} is the electro-optic tensor coefficient. Usable crystals also exhibit the piezoelectric effect to some degree, which has a small contribution to $\Delta\varphi$.

The interference intensity is given by

$$I = I_s + I_r + 2\sqrt{I_s I_r}\cos(\Delta\varphi), \tag{S6}$$

where I_s and I_r are the signal and reference beam intensity, respectively. Assuming $I_s = I_r$, then

$$\begin{aligned} I(x,y,t) &= 4I_r(x,y)\cos^2\left[\Delta\varphi(x,y,t)/2\right] \\ &= 4I_r(x,y)\cos^2\left[\frac{\pi}{\lambda}\left(\frac{x^2+y^2}{2R}+\frac{\lambda}{2}\right)+\varphi(x,y,L,t)\right] \\ &= 4I_0\exp\left(-\frac{x^2+y^2}{2w^2}\right)\times\cos^2\left[\frac{\pi}{2\lambda}\left(\frac{x^2+y^2}{R}\right)+\frac{\pi}{2}+\varphi(x,y,L,t)\right], \end{aligned} \tag{S7}$$

where I_0 is the laser central intensity, w is the Gaussian beam waist, R is the radius of the spherical wavefront, and L is the length of the LN crystal.

Supplementary Note 2 Double cable model of myelinated axons

The saltatory conduction of APs, which consist of active and passive current flows, along myelinated axons is commonly studied using the single cable equivalent circuit model, where the axon and myelin sheath are modeled as a tightly combined membrane that has no submyelin conduction pathways. However, the single cable model is not consistent with tracer and electron microscopy studies, which indicate a continuity between the extracellular medium and submyelin spaces[63]. Recently, Cohen et al. developed the double cable model[29], which addresses this discrepancy by considering the submyelin conduction in the periaxonal space that occurs in parallel to the axoplasmic conduction[64]. This submyelin current was included as an "extracellular" mechanism in the model in addition to the passive ion channels and active sodium, potassium, and calcium channels[29]. Here we employ the double cable circuit model to simulate the internodal current flows propagating in myelinated axons (Fig. 3b). We use the optimal parameters of axonal axial resistivity ($\mathrm{R_a}$), specific membrane resistance ($\mathrm{R_m}$) and capacitance ($\mathrm{C_m}$), specific myelin sheath resistance ($\mathrm{R_{my}}$) and capacitance ($\mathrm{C_{my}}$), periaxonal resistivity ($\mathrm{R_{pa}}$), and paranodal resistivity ($\mathrm{R_{pn}}$) as described in Supplementary Table 1.

Supplementary Table 1 Parameters of the double cable model

**Parameter**	$\mathbf{R_a}$ (Ω cm)	$\mathbf{R_m}$ (kΩ cm^2)	$\mathbf{C_m}$ (μF cm^{-2})	$\mathbf{R_{my}}$ (kΩ cm^2)	$\mathbf{C_{my}}$ (μF cm^{-2})	$\mathbf{R_{pa}}$ (GΩ cm^{-1})	$\mathbf{R_{pn}}$ (TΩ cm^{-1})	$\mathbf{s_{my}}$
**Value**	200	24.6	1.37	240	0.038	125	2.45	1.43

$\mathrm{s_{my}} = 1/0.7$ is a scale factor for myelin that accounts for the difference in surface area between the myelin sheath and myelin core. The specific surface resistance of extracellular space is $\mathrm{R_{my}}/\mathrm{s_{my}}$, and the specific surface capacitance of extracellular space is $\mathrm{C_{my}} \cdot \mathrm{s_{my}}$.

Supplementary Note 3 Mounting individual myelinated axons

Due to the difficulty in dissecting, isolating, and teasing individual myelinated axons from the *Xenopus laevis* sciatic nerve, we had less than a 40% success rate in observing the propagating internodal current flow with Diff-CUP. In most of the unsuccessful experiments with the myelinated axons, we observed no propagation profiles in both control and stimulus groups, which suggested that the axon had been damaged due to dissection failures, accidental contacts with tweezers or electrodes, unstable transfer, low environmental temperatures, or excessive stimulations[58–60].

We attempted to increase the success rate by building a customized nerve chamber made from a 1-mm thick transparent acrylic sheet (70 mm × 24 mm) with three 0.5-mm deep polished pools separated by a 0.5-mm gap to house the axon[60]. Vaseline was used for conductive insulation between different pools. However, the nerve chamber did not improve the success rate, since it required an additional step of transferring the teased axon from the dissection slide to the chamber, which further increased the chance of axon damage. Moreover, the acrylic sheet was prone to scratching by the electrodes, thus scattering the transmitted light for interferometric imaging. Therefore, we expect that the protocol for preparing individual axons could be further optimized to have less complexity, good transparency, and resistance to scratching, which would lead to an increased success rate.

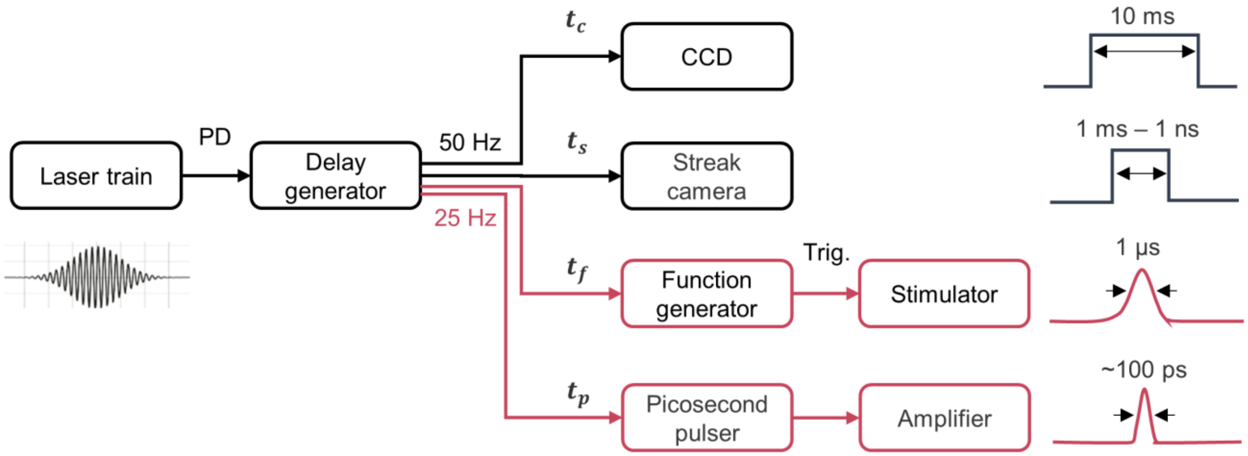

Supplementary Fig. 1

Synchronization of instruments in Diff-CUP. PD, photodiode; CCD, charge-coupled device camera; Trig, triggering signal. t_c, t_s, t_f, and t_p, delay for CCD, streak camera, function generator, and picosecond pulser, respectively. The laser pulses are downscaled to 50 Hz for triggering the CCD and the streak camera, and 25 Hz for the function generator and the picosecond pulser. The exposure time of the CCD is 10 ms. The time-shearing window of the streak camera is varied from 1 ms to 1 ns, depending on the duration and temporal resolution for Diff-CUP reconstruction. The stimulator triggered by the function generator provides 1 μs field stimulation to inject internodal current flows in myelinated axons. When delivered to the LN crystal, the amplified EMPs generated by the picosecond pulser have a pulse width of approximately 100 ps.

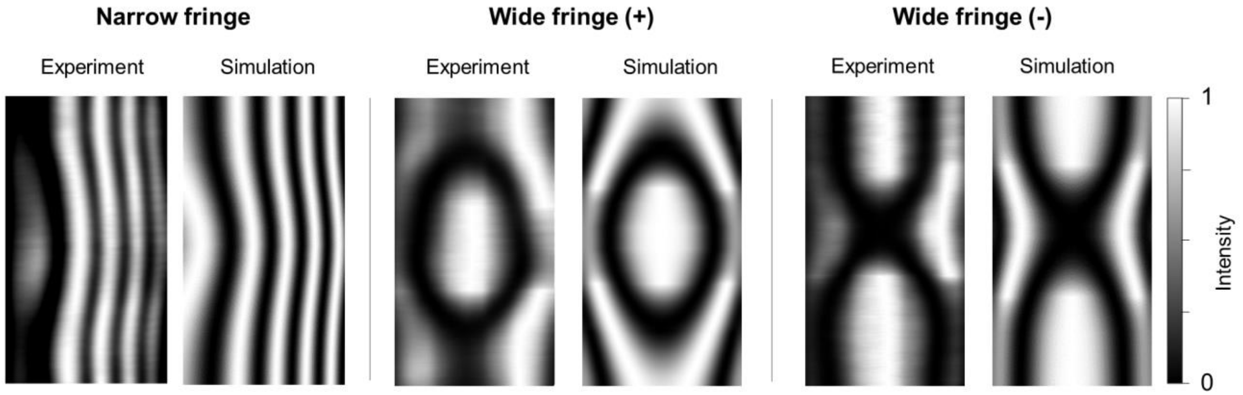

Supplementary Fig. 2

Experimental and simulated interferograms in different fringe modes. Phase changes resulted from a 2.6-ns EMP. From left to right: narrow fringe, wide fringe with positive phase change (+), and wide fringe with negative phase change (-). The wide-fringe mode could theoretically achieve a greater sensitivity; however, it was easily affected by vibration and hardly realized in axon interferometric imaging.

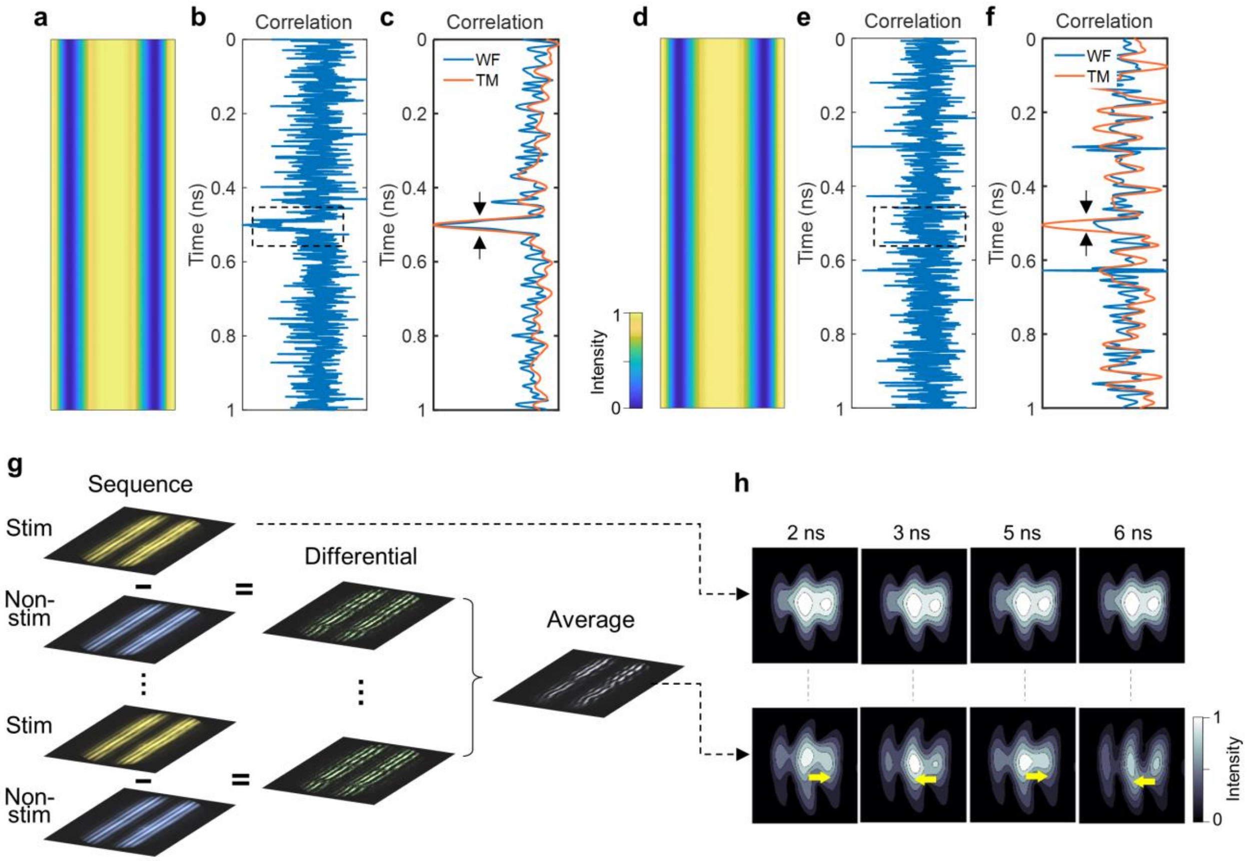

Supplementary Fig. 3

Processing of spatiotemporal interferograms. a, Simulated spatiotemporal interferogram of an EMP propagating in an LN crystal with zero-mean, 0.001-standard deviation Gaussian white noise added. **b**, Correlation processing of **a** reveals the pulse shape of the simulated EMP. Black arrows denote the pulse width of the EMP. **c**, Wavelet filtering (WF) and TM methods both reveal the pulse shape of the simulated EMP. Black arrows denote the pulse width of the EMP. **d**, Spatiotemporal interferogram identical to **a** but with zero-mean, 0.002-standard deviation Gaussian white noise added. **e**, Correlation processing of **d** cannot reveal the pulse shape of the simulated EMP. **f**, Only the TM method can reveal the pulse shape of the simulated EMP. Black arrows denote the pulse width of the EMP. **g**, Illustration of the differentially enhanced method. Subtraction is performed between two adjacent interferograms. **h**, Comparison of phase changes between conventional CUP and Diff-CUP. Yellow arrows indicate the phase shifts which cannot be observed by conventional CUP.

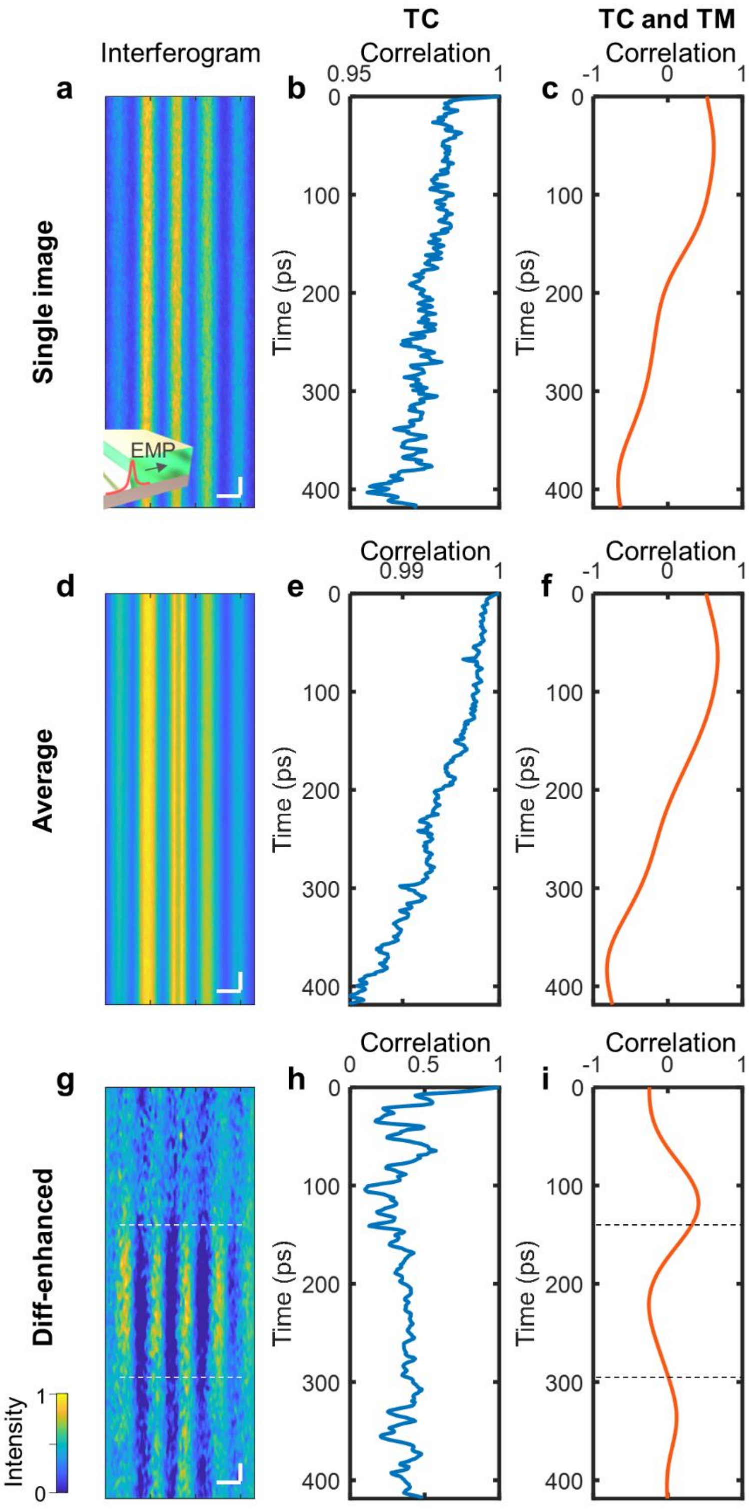

Supplementary Fig. 4

Comparison of different processing methods. For a weak EMP propagating in the LN crystal (inset of **a**), a single spatiotemporal interferogram (**a**) cannot be used to reconstruct the pulse, even

if it is processed through the TC (**b**) or both the TC and TM (**c**) method. Averaging 200 interferograms (**d**) and processing (**e**, **f**) also cannot reconstruct the pulse. The differentially enhanced method, i.e., averaging of 200 differential interferograms, (**g**) with TC processing (**h**) still cannot reconstruct the pulse. Only the differentially enhanced method combined with TC and TM processing (**i**) can reconstruct the pulse. White and black dashed lines denote the regions of the EMP. Horizontal scale bars, 300 μm. Vertical scale bars, 20 ps.

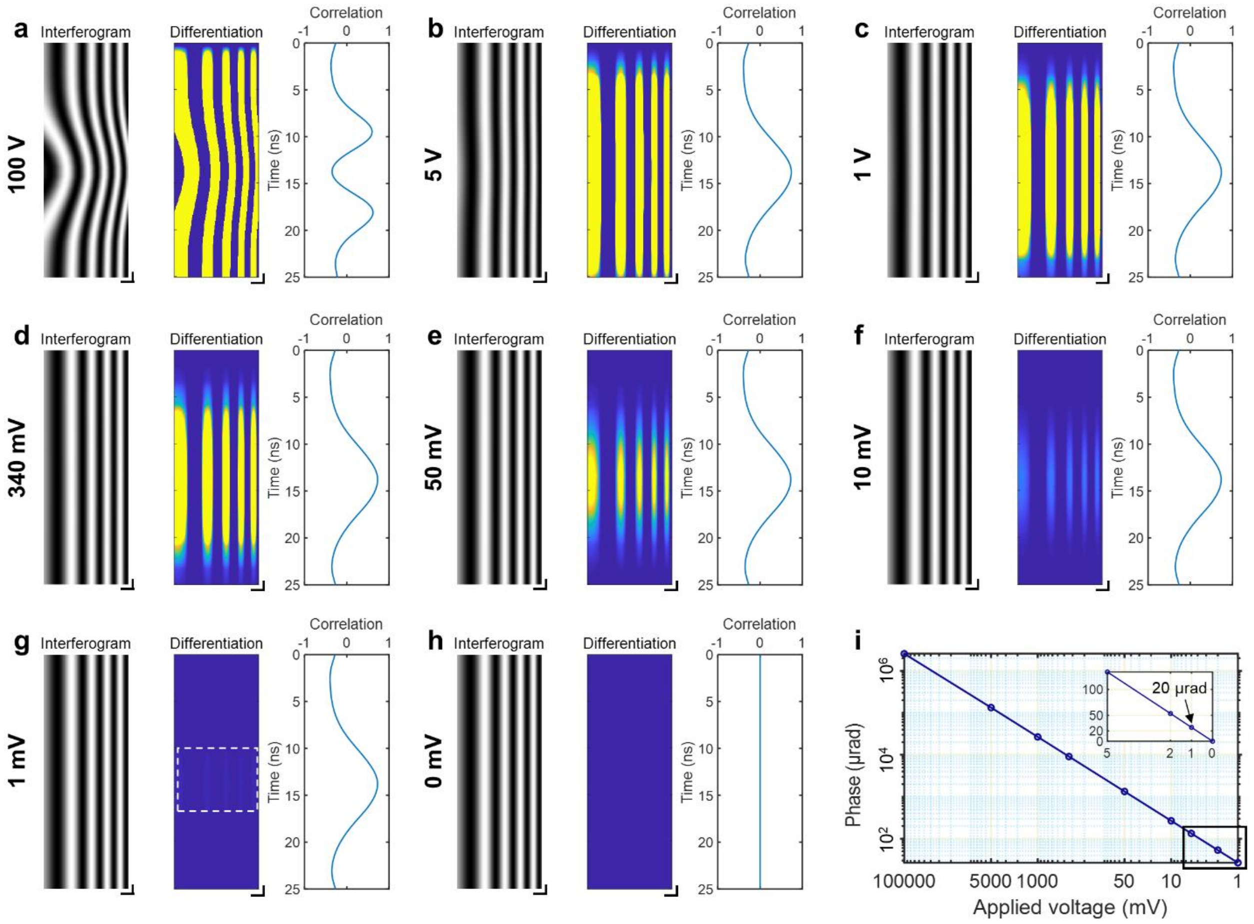

Supplementary Fig. 5

Simulation of the relationship between applied voltages and phase changes using the electric field and phase distribution model. a-h, Spatiotemporal interferograms of a 2.6-ns EMP with different peak voltages induced in the LN crystal, their differential interferograms, and the corresponding Diff-CUP reconstruction results (differential interferograms processed with the TC and TM methods). White dashed box denotes the region of the EMP with 1-mV peak voltage. **i**, Relationship between the EMP-induced phase change and the corresponding peak voltages. Inset, closeup of the region denoted by the black box. The minimum phase change, i.e., 20 μrad, corresponding to the 1-mV EMP is the phase sensitivity. Horizontal scale bars, 300 μm. Vertical scale bars, 1 ns.

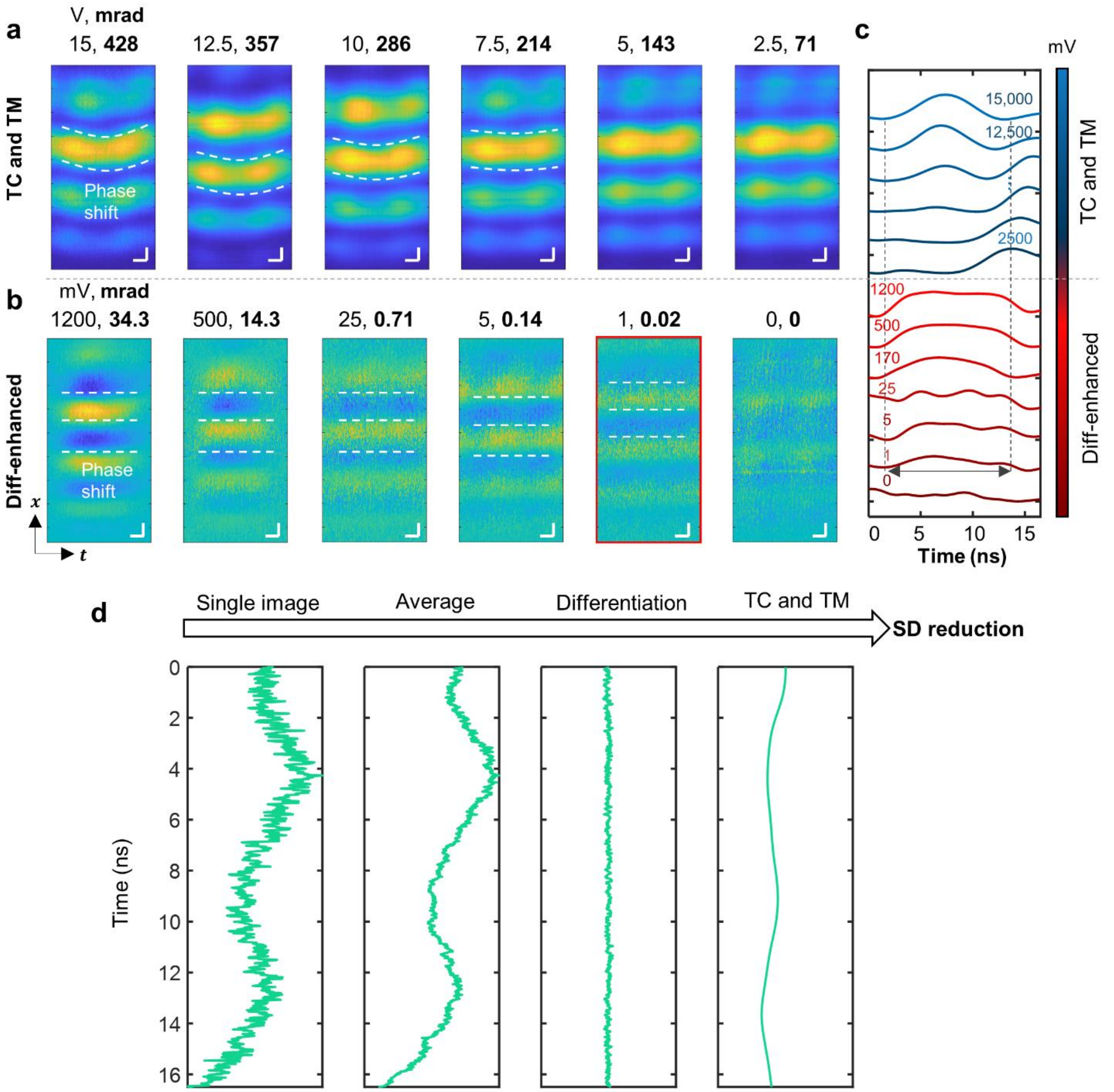

Supplementary Fig. 6

Reconstruction of EMPs using uncoded Diff-CUP. a, Spatiotemporal interferograms of a 2.6-ns EMP (peak voltages ranging from 30V to 5V) induced in the LN crystal, processed with the TC and TM methods. Here, unlike other spatial temporal interferograms in this paper, the horizontal and vertical axes denote time (t) and space (x), respectively, to align with the horizontal time axis in **c**. White dashed lines denote the regions of the EMP. Corresponding phase changes are shown in bold text. The large phase change can be identified without using the differential approach. **b**, Differential interferograms of the EMP (peak voltages ranging from 2400 mV to 0), processed

with the TC and TM methods. The small phase change can be observed due to the differential approach. Red box denotes the smallest phase change, i.e., 20 μrad, in response to the 1-mV EMP, which is the phase sensitivity. **c**, Diff-CUP reconstructed results of the interferograms in **a** and **b**. Each curve represents a reconstructed pulse shape with the corresponding EMP peak voltage shown above. Black dashed lines denote the region of the EMP. Black arrows denote the pulse width of the EMP. **d**, Experimental noise statistics when imaging EMPs following the Diff-CUP pipeline. From left to right: TC processing of a single interferogram without stimulation, TC processing of the average of N interferograms, TC processing of the average of N differential interferograms, TC and TM processing of the average of N differential interferograms (N = 200). Black hollow arrow indicates that the processing methods in the Diff-CUP pipeline reduce the standard deviation (SD) of the interferogram without stimulation. Vertical scale bars, 120 μm. Horizontal scale bars, 2 ns.

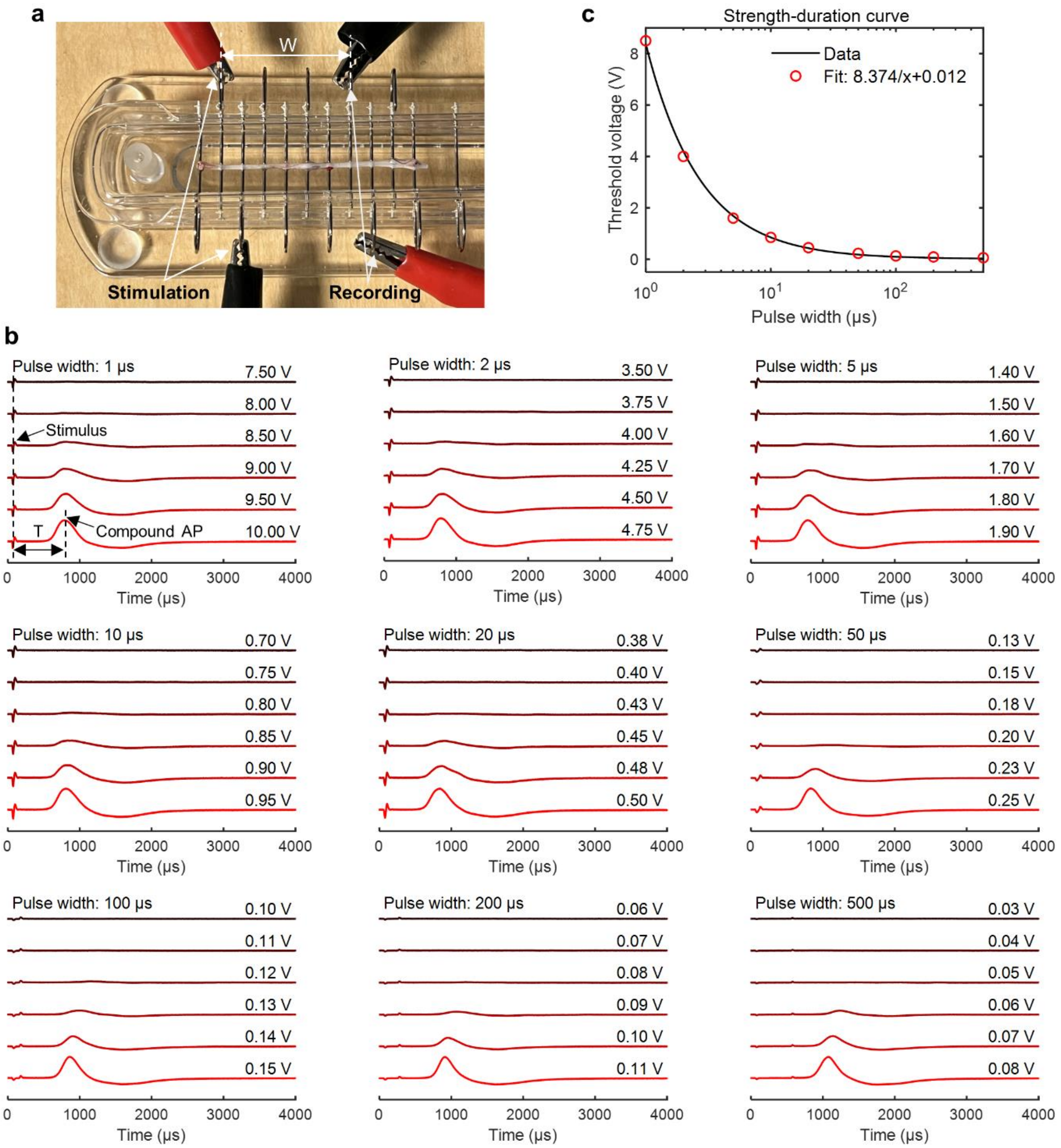

Supplementary Fig. 7

Electrical recordings of compound APs. a, Photo of the nerve chamber used for stimulating and recording compound APs from a dissected sciatic nerve bundle. W (≥ 2.5 cm) denotes the distance between the stimulating and recording electrodes. **b**, Compound APs recorded using the setup in **a** under different stimulating pulse widths and peak voltages. The stimulating pulses were generated by an isolated pulse stimulator. T denotes the time difference between the stimulus artifact and the compound AP. **c**, Strength-duration curve showing stimulation threshold voltages versus pulse widths required to elicit compound APs based on the recordings in **b**.

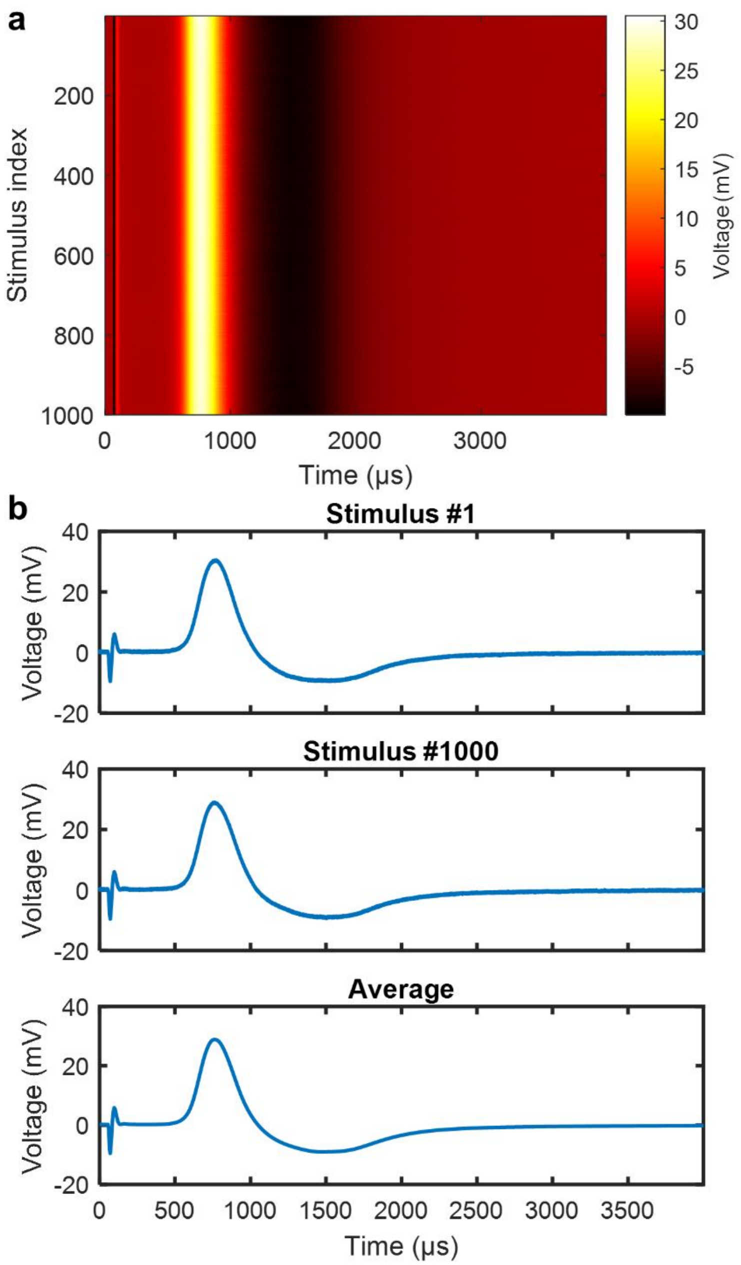

Supplementary Fig. 8

Verification of the lack of nerve damage through repeated reproduction of compound APs. **a**, 1000 compound APs recorded from repeated stimulations of 10-V, 1-μs pulses at 10 Hz. **b**, The first (top), last (middle), and average of all the compound APs in **a**.

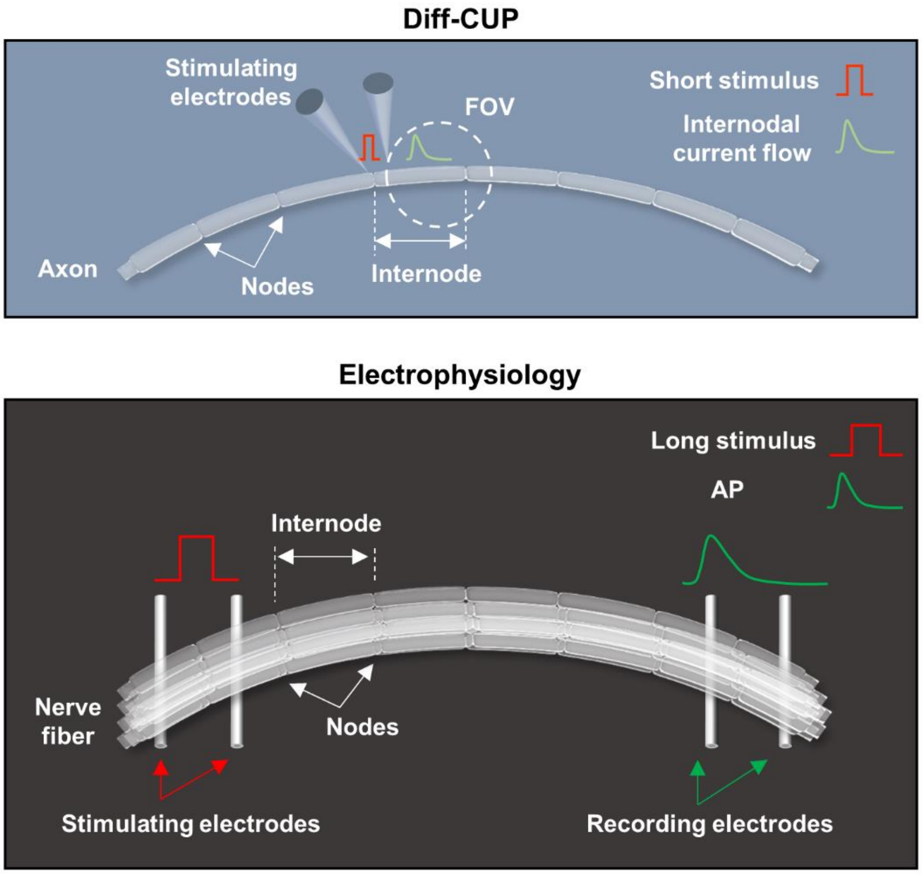

Supplementary Fig. 9

Schematics of the experimental setups used to inject internodal current flows. Top, in our Diff-CUP setup, short stimuli (1 μs) are used to inject passive current flows for propagation in a single internode (the same size as our FOV); bottom, in conventional electrophysiological setup, long stimuli (1–500 μs) are used to elicit APs for propagation across multiple nodes in the axon.

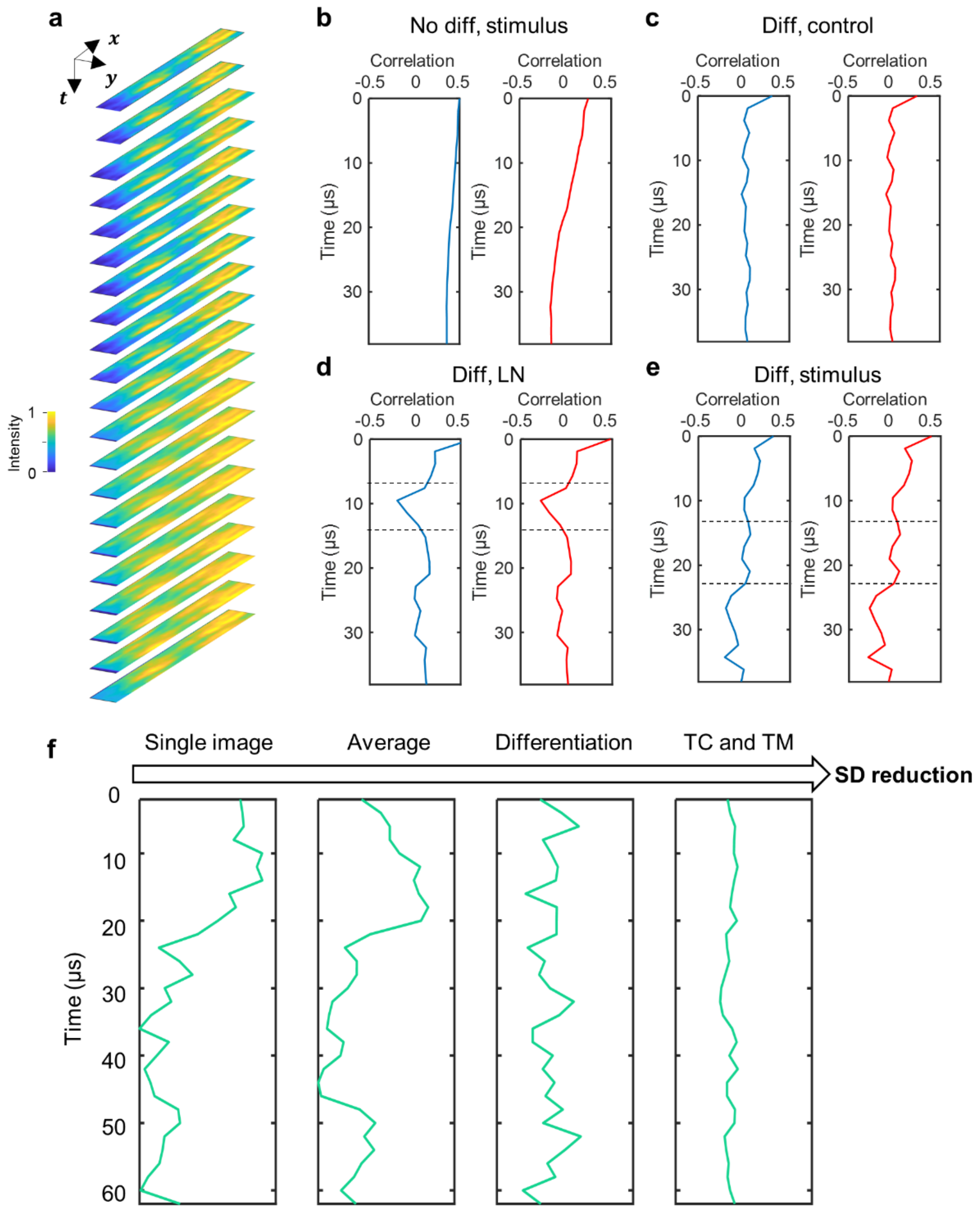

Supplementary Fig. 10

Reconstruction of internodal current flows using uncoded Diff-CUP. a, Spatiotemporal interferogram of a propagating internodal current flow in a myelinated axon rearranged in 3D. The vertical axis of the interferogram is expressed as $z = nd + y$, where n denotes the n-th axon image in the time series, and d represents the FOV in the y dimension. **b**, Blue and red curves show TC and both TC and TM processed results of the raw interferogram with stimulus. **c**, Processed results of the differential interferogram without stimulus (control group). **d**, Processed

results of the differential interferogram with a synchronous EMP induced in the LN crystal. Black dashed lines denote the duration of the EMP. **e**, Processed results of the differential interferogram with stimulus. Black dashed lines indicate the signal region of the AP. **f**, Experimental noise statistics in imaging internodal current flows following the Diff-CUP pipeline. From left to right: TC processing of a single interferogram without stimulation, TC processing of the average of N interferograms, TC processing of the average of N differential interferograms, TC and TM processing of the average of N differential interferograms. $N = 200$. Black hollow arrow indicates that the processing methods in the Diff-CUP pipeline reduce the SD of the interferogram without stimulation.

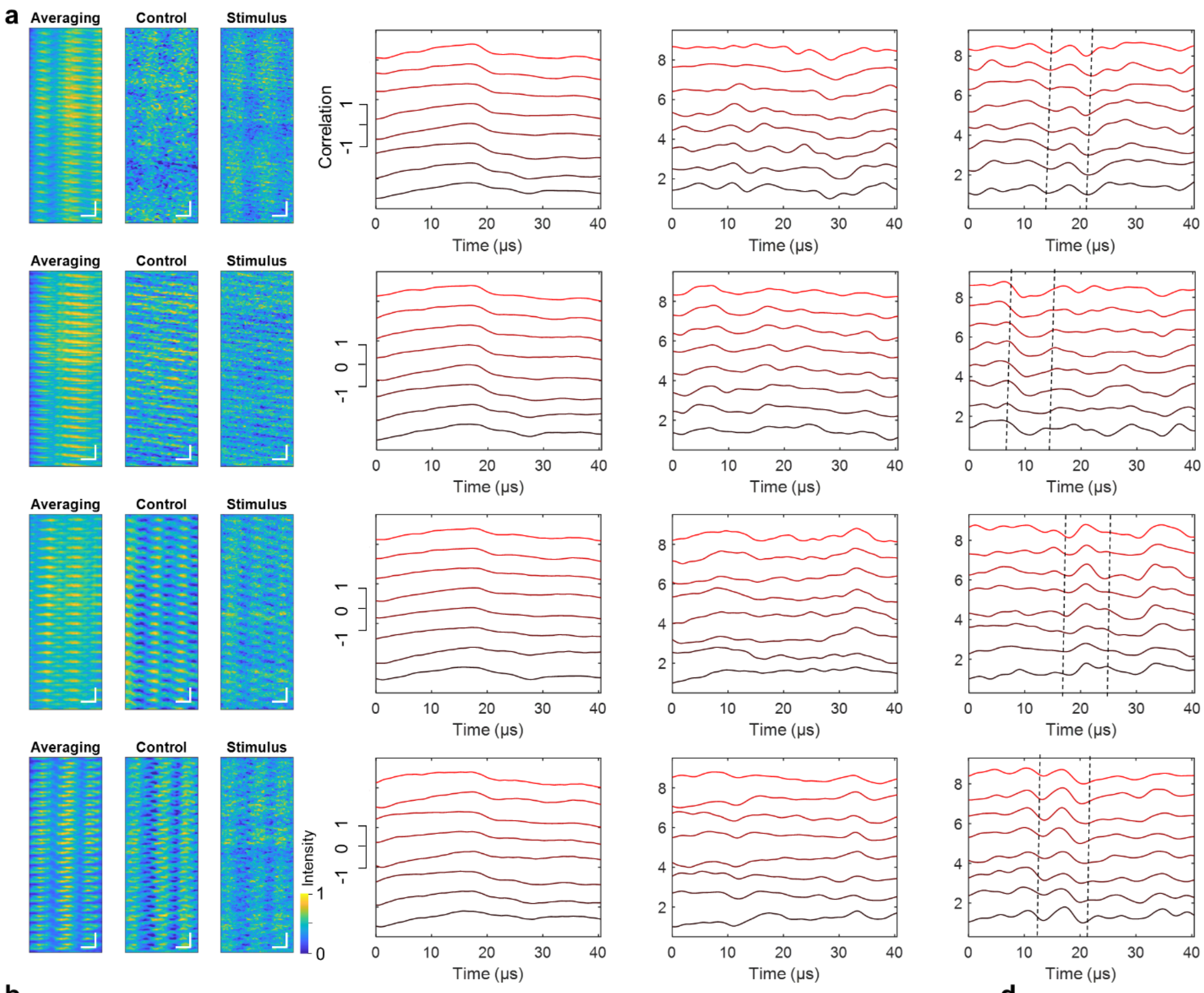

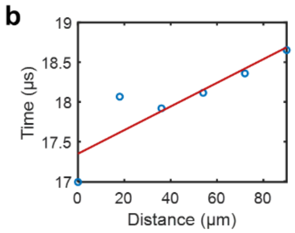

c

Axon #	1	2	3	4	5	6
Speed (m/s)	61.6	55.9	123.3	225	80.4	67.2

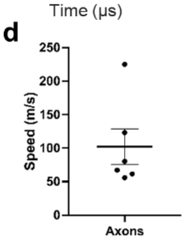

Supplementary Fig. 11

Conduction speed in myelinated axons extracted by uncoded Diff-CUP. a, Uncoded Diff-CUP reconstructions of propagating internodal current flows in myelinated axons acquired from four animals (each row representing an animal). The spatiotemporal interferograms captured by uncoded Diff-CUP (400 interferograms) under different conditions and their corresponding reconstructions (each correlation curve corresponds to a segment of the FOV) are shown on the left and right, respectively. Averaging, averaged results of unprocessed data; control, Diff-CUP processed results acquired without field stimulation; stimulus, Diff-CUP processed results

captured with field stimulation. Black dashed lines indicate the regions with propagating internodal current flows, which were identified by the similar shape of the peaks with correct temporal shifts that matched the simulated and reported speeds. Horizontal scale bars, 25 µm. Vertical scale bars, 3 µs. Segmented scale bars show the normalized correlation values **b**, Calculation of the conduction speed of an internodal current flow based on the temporal (peak time of the flow) and spatial (distance between the starting point to the segment's location within the FOV) locations of the propagating current flow, denoted as blue circles, in a group of correlation curves acquired with field stimulation. Red line is a linear fitting of the spatiotemporal locations of the propagating current flow, whose slope is used to calculate the conduction speed (67 m/s for the data shown here). **c**, Table showing conduction speeds of internodal current flows measured from six axons extracted from four animals. **d**, Scatter plot of the conduction speeds in **c**. Data are presented as the mean value (100 m/s) $\pm$ the standard error of the mean (26 m/s) (n = 6).

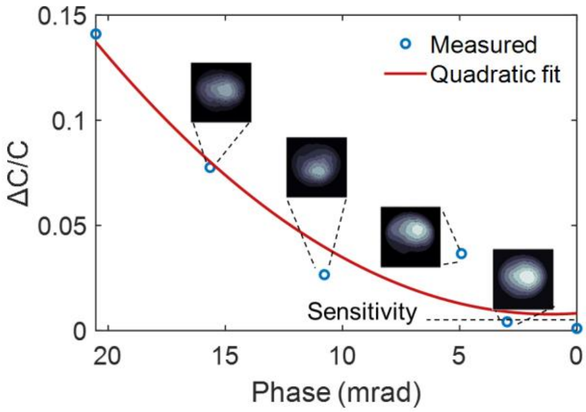

Supplementary Fig. 12

Quantification of phase sensitivity of coded Diff-CUP. When EMPs with different amplitudes are induced in the LN crystal, different levels of phase changes are imaged by Diff-CUP operating in the coded mode (shown as snapshots here), and different maximum correlation changes ($\Delta \mathrm{C}/\mathrm{C}$ with C equals to one) can be calculated. The phase sensitivity of coded Diff-CUP, quantified as the phase change corresponding to $\Delta \mathrm{C}/\mathrm{C} = 0$, is about 3 mrad.

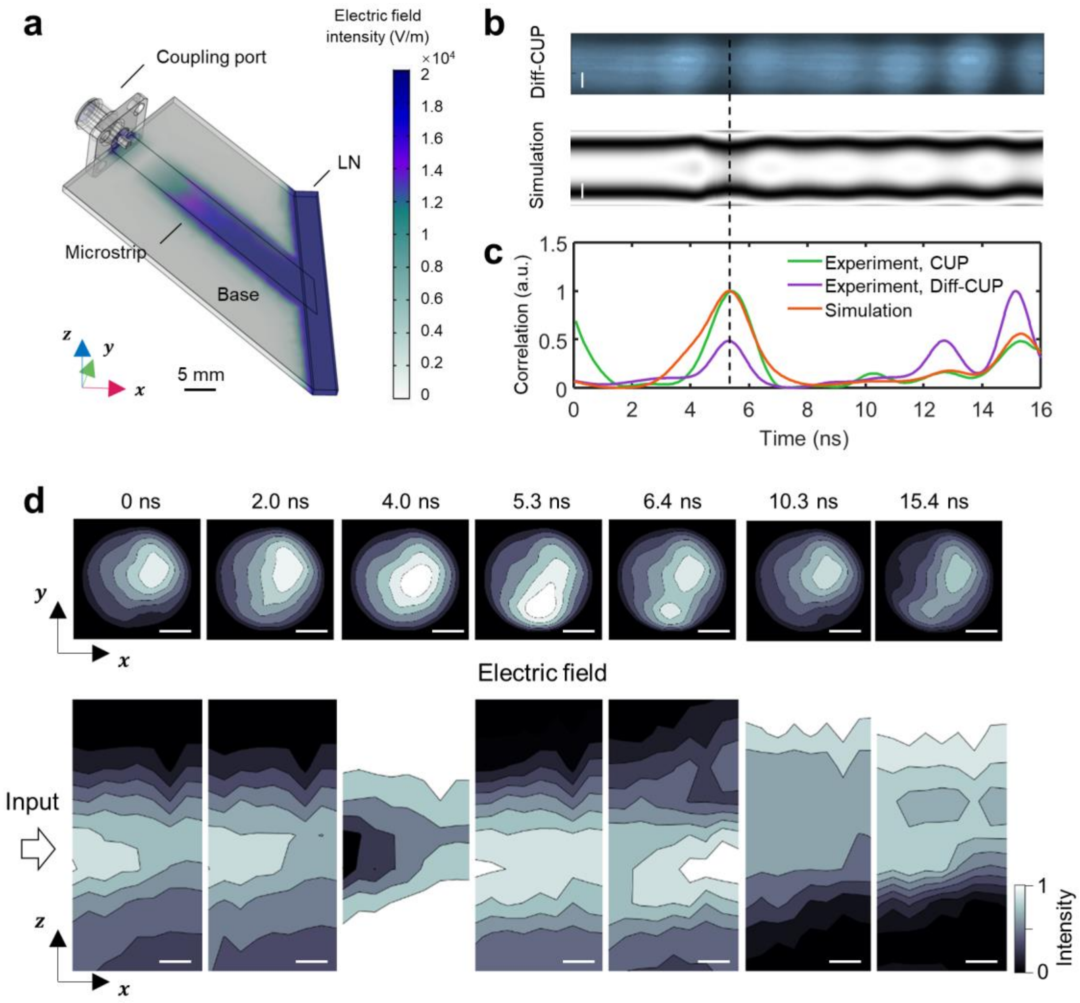

Supplementary Fig. 13

Coded Diff-CUP imaging and computational modelling of propagating EMPs. a, Finite element modeling of a 1-ns EMP propagating in the microstrip line and the LN crystal using the COMSOL software. **b**, Wide-fringe spatiotemporal interferograms of the propagating EMP acquired experimentally using Diff-CUP (top) or through simulation (bottom). **c**, Temporal correlations obtained from the conventional CUP, Diff-CUP, and simulation interferograms. Black dashed line indicates the main peak of the EMPs. **d**, Top, XY-plane snapshots of the phase change induced by the propagating EMP (launched from left to right) reconstrued by coded Diff-CUP. Bottom, XZ-plane snapshots of the corresponding electrical field distribution modeled using the COMSOL software. Scale bars, 500 µm.
