# Supplementary material for: Ultrafast and hypersensitive phase imaging of propagating internodal current flows in myelinated axons and electromagnetic pulses in dielectrics
Source: Nat Commun. 2022 Sep 6;13:5247. doi: 10.1038/s41467-022-33002-8 (PMC9448739; doi:10.1038/s41467-022-33002-8)
Supplement: Supplementary file 2 — Description of Additional Supplementary Files [file 41467_2022_33002_MOESM2_ESM.pdf]

Description of Additional Supplementary Files

Supplementary Video 1

Experiment and simulation of a reconstructed propagating internodal current flow in a myelinated axon at a frame rate of 20 MHz. The propagation reconstruction (normalized intensity) overlaid on top of a microscope image, and the red corresponds to the current flow. The simulated internodal current flow propagated faster initially due to the quickly accumulated ions in response to stimulus but slows down due to the attenuation of intermembrane potential difference. Scale bars, 20 µm.

Supplementary Video 2

Experimental propagating internodal current flows in five axons at a frame rate of 20 MHz. The propagation reconstructions (normalized intensity) overlaid on top of microscope images. Scale bars, 20 µm.

Supplementary Video 3

Comparison between the reconstructed movies (normalized intensity) of EMPs propagating in the LN crystal acquired by conventional CUP (left) and Diff-CUP operating in the coded mode (right) at 23 billion fps. Scale bar, 500 µm.

Supplementary Video 4

Reconstructed movie of a 150-ps EMP propagating in the LN crystal acquired by Diff-CUP operating in the coded mode at 209 billion fps. Shown below is the relative phase change induced by the EMP obtained by subtracting the first frame from each frame in the movie. Both were shown in normalized intensity. Scale bar, 500 µm.

Supplementary Video 5

Computational modeling of multiple nanosecond EMPs propagating in the microstrip line and the LN crystal at 5 billion fps using the COMSOL software.
