# Supplementary material for: Ultrafast and hypersensitive phase imaging of propagating internodal current flows in myelinated axons and electromagnetic pulses in dielectrics
Source: Nat Commun. 2022 Sep 6;13:5247. doi: 10.1038/s41467-022-33002-8 (PMC9448739; doi:10.1038/s41467-022-33002-8)
Supplement: Supplementary file 8 — Reporting Summary [file 41467_2022_33002_MOESM8_ESM.pdf]

nature portfolio

Corresponding author(s): Lihong V. Wang

Last updated by author(s): Aug 8, 2022

Reporting Summary

Nature Portfolio wishes to improve the reproducibility of the work that we publish. This form provides structure for consistency and transparency in reporting. For further information on Nature Portfolio policies, see our Editorial Policies and the Editorial Policy Checklist.

Statistics

For all statistical analyses, confirm that the following items are present in the figure legend, table legend, main text, or Methods section.

n/a	Confirmed	
☐	☒	The exact sample size (*n*) for each experimental group/condition, given as a discrete number and unit of measurement
☐	☒	A statement on whether measurements were taken from distinct samples or whether the same sample was measured repeatedly
☒	☐	The statistical test(s) used AND whether they are one- or two-sided<br>*Only common tests should be described solely by name; describe more complex techniques in the Methods section.*
☒	☐	A description of all covariates tested
☒	☐	A description of any assumptions or corrections, such as tests of normality and adjustment for multiple comparisons
☐	☒	A full description of the statistical parameters including central tendency (e.g. means) or other basic estimates (e.g. regression coefficient) AND variation (e.g. standard deviation) or associated estimates of uncertainty (e.g. confidence intervals)
☒	☐	For null hypothesis testing, the test statistic (e.g. *F*, *t*, *r*) with confidence intervals, effect sizes, degrees of freedom and *P* value noted<br>*Give P values as exact values whenever suitable.*
☒	☐	For Bayesian analysis, information on the choice of priors and Markov chain Monte Carlo settings
☒	☐	For hierarchical and complex designs, identification of the appropriate level for tests and full reporting of outcomes
☒	☐	Estimates of effect sizes (e.g. Cohen's *d*, Pearson's *r*), indicating how they were calculated

Our web collection on statistics for biologists contains articles on many of the points above.

Software and code

Policy information about availability of computer code

Data collection	Python 3.7.6, MATLAB 2018b (MathWorks, Inc.), NEURON simulation environment (v7.7), COMSOL Multiphysics (v5.5), and LabVIEW 2018 (National Instruments, Corp.)
Data analysis	Python 3.7.6, MATLAB 2018b (MathWorks, Inc.), NEURON simulation environment (v7.7), COMSOL Multiphysics (v5.5), and two-step iterative shrinkage/thresholding algorithm (v2). The data processing and image reconstruction methods are described in detail in the Methods.

For manuscripts utilizing custom algorithms or software that are central to the research but not yet described in published literature, software must be made available to editors and reviewers. We strongly encourage code deposition in a community repository (e.g. GitHub). See the Nature Portfolio guidelines for submitting code & software for further information.

Data

Policy information about availability of data

All manuscripts must include a data availability statement. This statement should provide the following information, where applicable:

- Accession codes, unique identifiers, or web links for publicly available datasets
- A description of any restrictions on data availability
- For clinical datasets or third party data, please ensure that the statement adheres to our policy

All data are available within the Article and Supplementary Information, or available from the corresponding author upon reasonable request.

Field-specific reporting

Please select the one below that is the best fit for your research. If you are not sure, read the appropriate sections before making your selection.

- [x] Life sciences
- [ ] Behavioural & social sciences
- [ ] Ecological, evolutionary & environmental sciences

For a reference copy of the document with all sections, see nature.com/documents/nr-reporting-summary-flat.pdf

Life sciences study design

All studies must disclose on these points even when the disclosure is negative.

Sample size	14 Xenopus laevis were used in the study. The choice of animal number was based on needs to experimentally image the propagation of internodal current flows along myelinated axons using our system. No statistical significance was quantified for this study.
Data exclusions	Due to the difficulty in dissecting, isolating, and teasing individual myelinated axons from the Xenopus laevis sciatic nerve, we had less than a 40% success rate in observing the propagating internodal current flow with Diff-CUP. Therefore, we excluded the failed axons from the results.
Replication	The reproducibility of the finding was evaluated via repeated measurements. We imaged propagating action potentials along six myelinated axons extracted from four animals. All attempts at replication were successful.
Randomization	The experiments were not randomized. Randomization was not relevant to this study because we are not comparing between samples.
Blinding	Blinding was not applicable during experiments and outcome assessment because we do not compare between samples or populations.

Reporting for specific materials, systems and methods

We require information from authors about some types of materials, experimental systems and methods used in many studies. Here, indicate whether each material, system or method listed is relevant to your study. If you are not sure if a list item applies to your research, read the appropriate section before selecting a response.

Materials & experimental systems

n/a	Involved in the study	
[x]	[ ]	Antibodies
[x]	[ ]	Eukaryotic cell lines
[x]	[ ]	Palaeontology and archaeology
[ ]	[x]	Animals and other organisms
[x]	[ ]	Human research participants
[x]	[ ]	Clinical data
[x]	[ ]	Dual use research of concern

Methods

n/a	Involved in the study	
[x]	[ ]	ChIP-seq
[x]	[ ]	Flow cytometry
[x]	[ ]	MRI-based neuroimaging

Animals and other organisms

Policy information about studies involving animals; ARRIVE guidelines recommended for reporting animal research

Laboratory animals	All myelinated axons were dissected from female 9+ cm Xenopus laevis (10-12 months old).
Wild animals	No wild animals were used in this study.
Field-collected samples	No field-collected samples were used in this study.
Ethics oversight	Ethics oversight was performed by the Institutional Animal Care and Use Committee of the California Institute of Technology.

Note that full information on the approval of the study protocol must also be provided in the manuscript.
